# Microfluidic devices for small-angle neutron scattering^1^

**DOI:** 10.1107/S1600576718007264

**Published:** 2018-06-01

**Authors:** Carlos G. Lopez, Takaichi Watanabe, Marco Adamo, Anne Martel, Lionel Porcar, João T. Cabral

**Affiliations:** a Department of Chemical Engineering, Imperial College London, South Kensington Campus, London SW7 2AZ, UK; b Institut Laue–Langevin, 71 avenue des Martyrs, 38042 Grenoble, France

**Keywords:** microfluidic devices, small-angle neutron scattering, lab-on-a-chip, closed-face polymer photolithography

## Abstract

A comparative examination is presented of materials and approaches for the fabrication of microfluidic devices for small-angle neutron scattering (SANS). Representative inorganic glasses, metals, and polymer materials and devices are evaluated under typical SANS configurations. Performance criteria include neutron absorption, scattering background and activation, as well as spatial resolution, chemical compatibility and pressure resistance, and also cost, durability and manufacturability. Closed-face polymer photolithography between boron-free glass (or quartz) plates emerges as an attractive approach for rapidly prototyped microfluidic SANS devices, with transmissions up to ∼98% and background similar to a standard liquid cell (*I* ≃ 10^−3^ cm^−1^). For applications requiring higher durability and/or chemical, thermal and pressure resistance, sintered or etched boron-free glass and silicon devices offer superior performance, at the expense of various fabrication requirements, and are increasingly available commercially.

## Abstract 2

A review of materials and fabrication methods provides recommendations for microdevices for small-angle neutron scattering.

## 1. Introduction   

Microfluidic devices can generate exceptionally precise flow fields and manipulate fluids within length scales from 1 mm to sub-micrometre (Squires & Quake, 2005 ▸), approaching dimensions characteristic of common soft-matter structures such as vesicles or aggregates. The time scales on which shear can be applied are commensurate with the relaxation times of many systems, including solutions of polymers or colloids. In this sense, the time and length scales of microfluidics and soft matter or complex fluids ‘share a common physicochemical parameter space’ (Bartolo & Aarts, 2012 ▸). Furthermore, microfluidic formulators enable the generation of vast sample arrays in segmented or continuous flows with minute sample volumes (pico- to nanolitres), valuable for the composition or phase mapping of mixtures, and they are amenable to coupling with scattering or diffraction (Zheng *et al.*, 2003 ▸; Zhou *et al.*, 2008 ▸; Schwemmer *et al.*, 2016 ▸; Pham *et al.*, 2017 ▸).

Small-angle neutron scattering (SANS) is a unique non-invasive probe for the structure and dynamics of soft matter. A variety of flow cells (Eberle & Porcar, 2012 ▸) have been employed to study the structure, rheology and kinetics of soft matter under flow, and representative examples are given in Table 1 ▸. The steady increase in SANS neutron flux at both reactor and pulsed sources, primarily due to improved neutron guides, and improvements in detector technology now enable microfluidic SANS experiments, opening a range of possibilities to interrogate simple and complex fluids (Lopez *et al.*, 2015 ▸; Adamo *et al.*, 2017 ▸, 2018 ▸).

While a number of microfabrication techniques for microfluidic SAXS (small-angle X-ray scattering) (Greaves & Manz, 2005 ▸; Ghazal *et al.*, 2016 ▸) have been developed (Shastry *et al.*, 1998 ▸; Stehle *et al.*, 2013 ▸; Merlin *et al.*, 2011 ▸; Møller *et al.*, 2013 ▸; Skou *et al.*, 2014 ▸; With *et al.*, 2014 ▸; Beuvier *et al.*, 2015 ▸; Rodríguez-Ruiz *et al.*, 2017 ▸), these are not directly transposable to neutrons. Neutrons interact with the atomic nucleus itself, which varies significantly between elements and isotopes. In addition to the design and fabrication of traditional SANS cells, which are well established and have been recently reviewed (Bailey, 2003 ▸; Barker & Mildner, 2015 ▸), microfabrication methods have additional requirements (notably feature resolution) and generally benefit from rapid prototyping techniques. To date, there has not been a systematic examination of microdevice fabrication materials and methods for microfluidic SANS, which is the purpose of this work.

This paper is organized as follows. We first illustrate recent developments in microfluidic SANS, applied to the phase mapping and flow processing of complex fluids. In order to rationalize the materials and microfabrication evaluation, the basic principles of SANS are outlined, followed by a survey of the suitability of different materials and methods for microdevice fabrication. A number of approaches are considered, and recommendations are made for three that show particular promise. We conclude with an assessment of the compatibility of various systems with microfludic SANS, estimating the feasibility of experimental measurements.

## 2. Opportunities for microfluidics and SANS   

**Figure 1:**
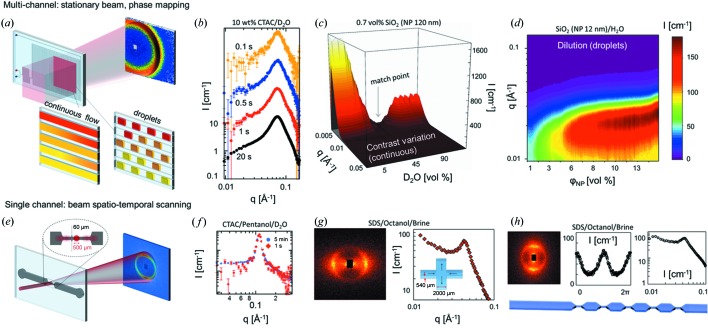
Configurations of microfluidic SANS for phase mapping (top row) and flow processing (bottom row). (*a*) Schematic of a continuous-flow and a droplet mixer, where a large (∼10 mm) neutron beam (shown in red) illuminates a number (10–20) of microchannels. (*b*) Patterns of radially averaged scattering intensity of a surfactant solution at acquisition times down to 0.1 s (data shifted vertically for clarity). (*c*) Rapid contrast variation with a nanoparticle suspension in 120 steps of 10 s at a flow rate of 0.1 ml min^−1^ (Adamo *et al.*, 2017 ▸). (*d*) Serial dilution in droplet microfluidics of a nanoparticle suspension (Ludox HS-40) in 120 steps of 5 s at a flow rate of 0.075 ml min^−1^ with a fluorinated oil carrier [adapted from Adamo *et al.* (2018 ▸) by permission of The Royal Society of Chemistry]. (*e*) Contraction–expansion geometry with a 500 µm neutron beam scanning the flow field. (*f*) Scattering intensity of a CTAC/pentanol/D_2_O mixture at 5 min and 1 s acquisitions. (*g*) Two-dimensional and radially averaged scattering of SDS/brine/octanol in an opposing jet (extensional) geometry and (*h*) through the first constriction of the illustrated device; adapted with permission from Lopez *et al.* (2015 ▸) under a Creative Commons Attribution 4.0 International License, https://creativecommons.org/licenses/by/4.0/.

Fig. 1 ▸ illustrates current capabilities of microfluidic SANS, for both equilibrium (top) and non-equilibrium (bottom row) studies of simple and complex fluids. The former include mapping of the composition space of mixtures to establish thermodynamic phase diagrams, and determination of the shapes and interactions of objects (colloids, micelles, aggregates *etc.*) in solution. These experiments are generally implemented by continually varying input compositions and acquiring SANS data at a fixed position in a microdevice, often overilluminating several channels, in both continuous (Adamo *et al.*, 2017 ▸) and droplet flows (Adamo *et al.*, 2018 ▸), as shown in Fig. 1 ▸(*a*). In these experiments, a square beam of 1 × 1 cm was employed, illuminating ∼16 channels. The beam illuminates a location along the microdevice selected to ensure mixing of various inputs but limited compositional dispersion, defining a (time-varying) system composition with prescribed precision. Given the comparatively large sample volume probed, sub-second acquisition times are easily attainable, as illustrated with a micellar solution in Fig. 1 ▸(*b*), since the effective sample volume reduction is modest. For instance, the illuminated channel area is typically 50–70% of the beam footprint (the remainder being occupied by the device matrix), and microchannel depths are typically 100–1000 µm. As a result, sample volumes are approximately 5–50% of those of a common liquid cell, requiring a modest adjustment of SANS measurement times. Rapid and precise contrast matching measurements, and systematic dilution or composition scans in ∼10 min (total) time scales, are readily possible (Figs. 1 ▸ *c* and 1 ▸ *d*), circumventing the traditional, sequential and time-consuming preparation of discrete samples, loading of cells and use of sample changers. Further, microfluidic SANS also allows adaptive scanning of parameter space (Adamo *et al.*, 2017 ▸) and possible feedback and optimization loops. In this mode, the device functions as an automated formulator, removing the sample making/loading/washing bottleneck.

Microfluidic SANS also provides unique opportunities in flow-SANS, to elucidate the mechanisms and kinetics of molecular and mesoscopic processes underpinning the flow response, assembly and metastability in complex and biological fluids. The approach is illustrated in the bottom row of Fig. 1 ▸. These studies often benefit from the rapid prototyping of microdevices to generate a variety of precise flow fields of tunable type and magnitude, for instance in the microfluidic four-roll mill (Hudson *et al.*, 2004 ▸; Lee *et al.*, 2007 ▸), and emulate flows relevant to manufacturing processes or porous media (Vasudevan *et al.*, 2010 ▸) as well as to fundamental rheology. In order to spatio-temporally probe the response of a complex fluid to a given flow field, the beam footprint should be small ‘enough’ to enable mapping by scanning device positions under steady-state conditions, employing an *xyz*θ stage. Neutron beams of typically hundreds of micrometres are employed, and sub-beam-footprint resolution is possible. Given the strong reduction in flux, this approach is only compatible with strongly scattering (*e.g.* highly ordered) fluids. Fig. 1 ▸(*e*) illustrates a contraction–expansion geometry for which scattering from a single ∼50 µm channel was attainable with a concentrated surfactant solution, shown in Fig. 1 ▸(*f*), and acquisition times down to 1 s per spectrum. For instance, cross-slot (and four-roll mill) and periodic constriction geometries, shown in Figs. 1 ▸(*g*) and 1 ▸(*h*), can be fabricated and the effect of flow quantified, beyond the traditional flow geometries accessible, as introduced in Table 1 ▸. Data analysis and interpretation must take into account the fluid velocity profile (*e.g.* parabolic or plug flow) within the microchannel, which normally requires ancillary flow measurements (Martin *et al.*, 2016 ▸; Poulos *et al.*, 2016 ▸; Weston *et al.*, 2018 ▸). Since multiple shear rates are effectively weighted in the data, systematic variation of channel geometry and flow rate provides new opportunities for rheo-SANS.

Equipped with the ability to label multicomponent systems selectively, we expect microfluidic SANS to build upon the accomplishments of microfluidic SAXS over the past two decades, for instance in high-throughput screening (Toft *et al.*, 2008 ▸), fast transformation kinetics (Pollack & Doniach, 2009 ▸; Graceffa *et al.*, 2013 ▸), flow processing (Lutz-Bueno *et al.*, 2016 ▸; Silva, 2017 ▸) and (bio-)molecular assembly (Koester & Pfohl, 2012 ▸; Bretagne *et al.*, 2017 ▸).

## 3. Coupling microfluidics and SANS   

SANS probes spatial correlations between ensembles of atomic nuclei on the nanoscale, typically covering a wavenumber range of 0.001 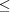 *q* 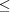 0.5 Å^−1^, corresponding to length scales (*d* ≃ 2π/*q*) of 1–500 nm. Experiments measure the differential macroscopic scattering cross section of the sample, a product of the form factor *P*(*q*) and the structure factor *S*(*q*):
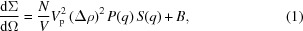
where *N*/*V* is the number density, *V*_p_ is the volume of a solute particle, (Δρ)^2^ is a contrast factor and *B* is the scattering ‘background’. *P*(*q*) contains information about the solute’s shape and size and *S*(*q*) about the spatial correlations between individual scattering species. Δρ can be effectively tuned by selective deuteration, enabling the direct determination of *P*(*q*) in concentrated or inter-penetrating systems. Given the number of excellent introductions to SANS and data analysis, we concentrate here on microdevice fabrication aspects.

### 3.1. Microfluidic requirements for SANS   

Microdevices for SANS experiments should meet a number of requirements. In short, they must exhibit (i) low neutron absorption; (ii) weak scattering in the experimental wavenumber *q* range relevant for SANS (approximately 0.001–0.5 Å^−1^) compared with the samples of interest; (iii) low neutron activation and/or short half-life decay; (iv) chemical compatibility with the sample and ability to withstand the required pressure, typically up to 1–10 bar (but potentially higher; 1 bar = 100 000 Pa), without deformation, as required for absolute SANS intensity calibration; (v) feature resolution in the range ∼1–1000 µm; and (vi) compatibility with rapid prototyping techniques (two and three dimensional) if flexible channel design is necessary. Specific applications may have additional requirements, such as oxygen permeability (*e.g.* for biological samples), surface functionality for prescribed wetting in multiphase flows, or control wall slip.

### 3.2. Neutron beam illumination, diaphragms and flux   

**Figure 2:**
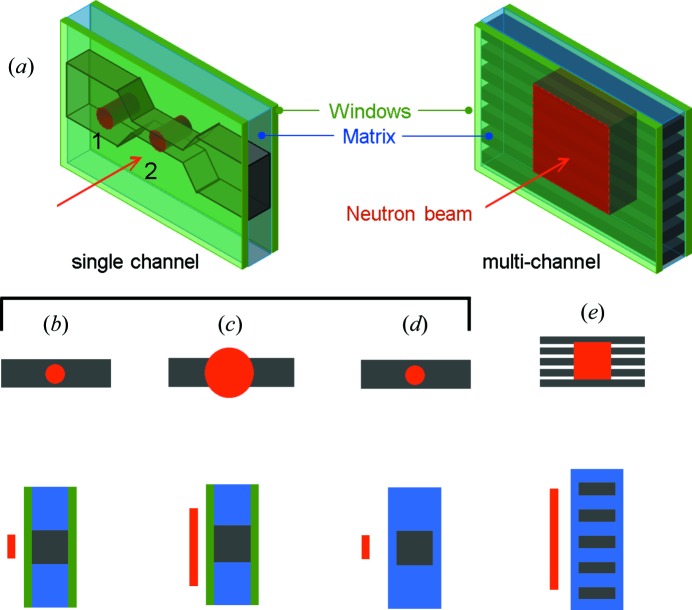
(*a*) Schematic of microdevice and beam configurations for (left) single-channel mapping with a beam of ∼100–1000 µm footprint and (right) overillumination of several channels, with a beam of ∼10 mm footprint. Representative configurations for the beam footprint and channel are given in panels (*b*)–(*e*), in both front view (top row) and cross section (bottom row).

Fig. 2 ▸ depicts representative illumination configurations for single-channel scanning and channel overillumination, noting that the beam may impinge not only on the microchannel window and sample, but also on the device matrix and/or several microchannels. These non-uniform cross sections, often involving several materials, have significant consequences for data reduction, which becomes non-standard. While a first-order correction simply accounts for the illuminated sample volume, accurate calibration (in relative or absolute units of cm^−1^) must take into account the various contributions to scattering, transmission and absorption from the microdevice components. A detailed description of the data reduction and calibration steps, including useful simplifications and assumptions, is presented in the supporting information.

At high scattering angles, additional geometric data corrections may be necessary since neutrons scattered close to the edge of a channel will pass through the channel matrix before reaching the detector, and are thus attenuated or scattered in a non-trivial manner. As a rule of thumb, the ratio of channel depth *d* to width *w* (or beam diameter, whichever is smaller) should satisfy 4*W*/*D* 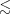 *w*/*d*, where *D* is the sample-to-detector distance and *W* is the maximum distance between the beam centre and the edge of the detector. For a typical high-*q* setup (*D* ≃ 1.5 m, *W* ≃ 1 m), *d*/*w* greater than 1–3 should be used. A full offset of the detector (often used to enlarge the *q* window) can increase this requirement by a factor of 2.

Neutron focusing optics (*e.g.* using MgF_2_ lenses) generally focus the beam at the detector to access lower *q* values, as required for very small angle scattering, vSANS; focusing at the sample is not suitable as a strategy to decrease the beam size for microfluidics owing to the accompanying increase in beam divergence, which in turn restricts the accessible low-*q* range (Mildner, 2014 ▸). Instead, slits or diaphragms are usually placed as close as possible to the microdevice in order to limit the beam footprint, divergence and air scattering. For a conventional SANS measurement, large circular apertures (∼12 mm in diameter) made of absorbing materials (Cd, Gd, enriched hot-pressed ^10^B_4_C) are employed to maximize the illuminated area and thus the scattering signal. Reducing the diameter of the diaphragm, and/or altering its shape, is thus needed to accommodate small samples. Evidently, reducing the beam footprint also reduces the neutron flux, in near proportionality to the area. For instance, reducing a circular beam of 12 mm diameter to 100 µm decreases the neutron flux by ∼10^6^, limiting the feasibility of some flow-mapping experiments, such as those shown in Fig 1 ▸(*e*). Generally, enriched hot-pressed ^10^B_4_C is preferred as a manufacturing material as it contains no hydrogen and produces many fewer γ-rays than the Cd or Gd counterparts. These apertures are usually 2 mm thick and cut by electro-erosion, as ^10^B_4_C is an extremely hard and brittle material. Slit apertures as small as 50 µm can be made to increase the spatial resolution and enable fine scanning. Furthermore, rectangular slits or multi-slit masks (registered with microchannels) can be desirable to increase the illuminated area and thus the signal and to minimize background scattering, although this requires precise alignment and positional stability.

We next consider different materials and techniques commonly used in microfabrication and their compatibility for microfluidic SANS, accompanied by representative SANS and transmission measurements.

## 4. Materials and methods   

### 4.1. SANS setup   

Neutron scattering measurements were performed on beamline D22 at the ILL reactor (Institut Laue–Langevin, Grenoble, France) and on the LOQ beamline at the ISIS pulsed source (Rutherford Appleton Laboratory, Oxfordshire, UK). On D22, the wavelength was fixed at a representative λ = 6 Å, and three sample-to-detector distances were used (1.2, 2.8 and 5.6 m), yielding a *q* range of 0.009 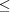 *q* 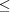 0.6 Å^−1^. Acquisition times ranged from 2 to 7 min for the high- and low-*q* configurations, respectively. The LOQ data were acquired with a polychromatic incident beam of λ = 2–10 Å and (fixed) 4 m distance, resulting in a *q* range of 0.009 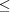 *q* 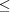 0.249 Å^−1^.

### 4.2. Materials   

Borosilicate cover slips (thickness *t* ≃ 140 µm) and soda lime glass slides (*t* ≃ 1 mm) were purchased from Fisher. Quartz slides (*t* ≃ 0.44 mm) and fused silica slides (*t* ≃ 1.1 mm) were purchased from H. Baumbach and Co. Ltd. Silicon wafers (*t* ≃ 0.34 mm, 〈100〉, single-side polished) were purchased from Si-Mat (Germany). A poly(dimethylsiloxane) (PDMS) film (*t* ≃ 0.6 mm) was produced by mixing Sylgard 184 elastomer with a curing agent (both obtained from Dow Corning) in a 10:1 weight ratio. The film was then degassed and cured at 348 K for 1 h. Hydrogenated and deuterated polystyrene (PS) and hydrogenated poly(methyl meth­acrylate) (PMMA) films (*t* ≃ 1 mm) were pressed from standard grades. A thiolene film (*t* ≃ 0.54 mm) was prepared by photocuring the optical adhesive Norland NOA-81 (purchased from Edmund Optics) with UV light. Kapton film (*t* ≃ 0.09 mm) was obtained from Müller Ahlhorn (Germany). An aluminium slab (*t* ≃ 1.2 mm) was obtained from the Imperial College London workshop; its scattering pattern was close to that of commercial (∼4 µm thick) aluminium foil.

Microfluidic experiments with polymer/glass (Lopez *et al.*, 2015 ▸) and glass devices (Adamo *et al.*, 2017 ▸, 2018 ▸) have been reported in detail. The choice of pumps is discussed in the supporting information.

## 5. Materials for microfabrication and experimental SANS evaluation   

### 5.1. Preliminary considerations   

As a reference, a typical SANS liquid cell, for example the ubiquitous Hellma QS series (made of two 1 mm thick windows of Suprasil quartz), exhibits a neutron transmission of ∼96% (at λ = 6 Å) and a scattering intensity of ∼5 × 10^−3^ cm^−1^. Fig. S4 in the supporting information summarizes the neutron absorption, transmission and scattering intensity for the most abundant elements, excluding rare earths, noble gases and highly reactive elements, but including major industrial elements and common precious metals (Ag and Au). Most low atomic number (*Z* 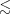 40) elements yield minimal neutron absorption, except for lithium, boron and cobalt. Hydrogen has a large incoherent cross section and scatters strongly, so its content must be minimized. In addition, crystalline materials with characteristic repeat distances *d* 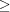 12–15 Å, and thus exhibiting pronounced structural peaks (at q* = 2π/*d*), should be avoided. Polycrystalline materials will also scatter in the low-*q* region owing to grain boundary interfaces (generally isotropically). Otherwise, a range of elements and materials would appear to yield reasonably high-quality SANS cells (*T* 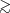 96%) in the high-*q* region, in particular for microdevices with sufficiently thin windows (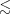
100 µm).

### 5.2. Transmission   

**Figure 3:**
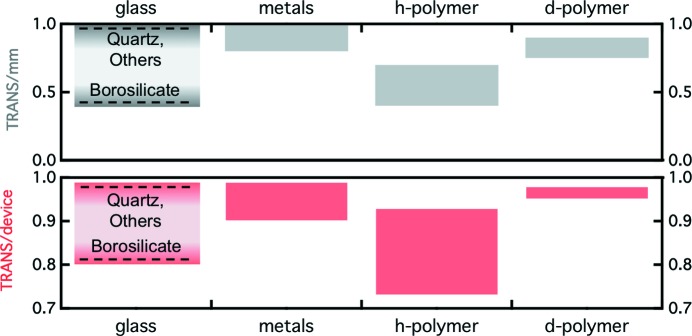
The range of neutron transmissions for different classes of material. The top panel shows the transmission (*T*) per 1 mm of material, and the bottom panel shows the estimated transmission of a microdevice with a likely thickness *t*_D_ (detailed in the text).

Fig. 3 ▸ shows the transmission for different classes of mat­erials used in the fabrication of microdevices, and specific values are compiled in Table S2 in the supporting information. In addition, the thickness required for microfabrication must be considered and thus we also estimate (bottom row) the transmission ranges of corresponding devices. For the different glasses, we assume the device thicknesses of our fabricated devices described in §6; for polymers such as PS, PMMA and bisphenol-A polycarbonate (PC), we assume two windows of 200 µm each (Metwally *et al.*, 2012 ▸; Dhouib *et al.*, 2009 ▸); and *t* = 250 µm for thiolene (Brennich *et al.*, 2011 ▸).

Inorganic glasses generally show high transmission, except borosilicate glass, for which thin (∼100 µm) windows are thus required. Polymers show a transmission of ∼0.6 ± 0.1 for 1 mm thick slabs, which translates to device transmissions of ∼0.8. Kapton devices with thin walls (5–50 µm; Evans *et al.*, 2007 ▸; Trebbin *et al.*, 2013 ▸; Catalano *et al.*, 2014 ▸) exhibit high transmissions, of the order of that of a Hellma cell. Deuterated polymers would also yield high transmissions for a typical thickness of 200 µm but are comparatively costly. Materials such as silicon and aluminium, with minimal neutron absorption (even for a thickness ∼1 cm), yield effectively 100% transmission for microdevices. Nickel, although a strong absorber, can be used to fabricate extremely thin devices (2 × 10 µm), thus yielding a transmission of ∼1. Taking the high transmission *T* = 96% of a Hellma cell as a reference, the window thicknesses required for a microdevice of equivalent transmission are compiled in Table S2 in the supporting information. Despite the significant differences in transmission, no class of material emerges as incompatible for microdevices for SANS.

### 5.3. Neutron activation   

Prolonged exposure to neutrons may induce considerable radioactivity in materials, which must decay to safe levels to enable handling, for instance when changing samples or upon completion of an experiment. Low activation and/or a short decay half-life are thus desirable for a microdevice material. While elements H, C, N and O, constituents of polymeric materials, are intrinsically safe, 1 g of Al, sapphire (Al_2_O_3_) or titanium will typically decay to safe levels within approximately 1 h, and the same mass of Si or SiO_2_ (*e.g.* quartz or fused silica) requires approximately 1 d after a reference 10 h exposure at 10^8^ neutrons cm^−2^ s^−1^. By contrast, Cu would require a few days, Zn and Fe a few years, and Ni and Co decades, under identical conditions (NIST, 2017 ▸). These estimates correspond to thicknesses of approximately 1 mm (ρ ≃ 10 g cm^−3^) to 10 mm (ρ ≃ 1 g cm^−3^) and a beam footprint of 1 cm^2^. Evidently, sufficiently thin metal sheet windows with a low mass (if compatible with microfabrication) and smaller beams will eventually yield lower or even imperceptible radioactivity. A configuration with a beam area of 0.25–1 mm^2^ impinging upon two metal windows of 10–50 µm thickness (Chandrasekaran *et al.*, 2003 ▸; Lang *et al.*, 2011 ▸) results in an irradiated mass of 0.05–1 mg of material (using ρ ≃ 10 g cm^−3^), which yields an acceptable decay time for a range of metals, as illustrated in Table 2 ▸.

The above estimates for 10 h exposure, albeit much longer than a single-spectrum acquisition, are reasonable for a series of microfluidic SANS experiments. While the beam is generally open throughout a SANS experiment, it is recommended that the beam is shut off during down-time (*e.g.* flow stabilization or cleaning) to minimize neutron exposure.

### 5.4. Scattering ‘background’: glasses, polymers, metals and ceramics   

#### 5.4.1. Glass and silicon   

A wide range of microfluidic devices are made of glass, quartz and silicon (Dittrich *et al.*, 2006 ▸; Iliescu *et al.*, 2012 ▸), which can be fabricated by machining and etching techniques, depending on the features required and any cost or time limitations (Lee & Sundararajan, 2010 ▸). Glass and silicon have excellent chemical resistance to most common solvents, as well as good thermal stability, optical transparency (in the case of glass) and Young’s modulus in the range of tens of GPa (Lee & Sundararajan, 2010 ▸). Quartz is a standard material for SANS cells, and it therefore seems like an obvious choice for the fabrication of neutron-compatible microdevices. The compositions of different glasses are compiled in Table S1 in the supporting information.

**Figure 4:**
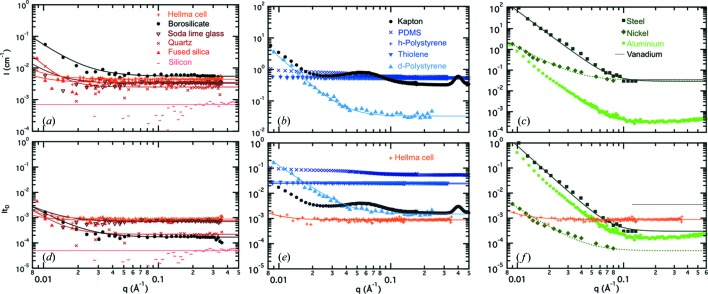
Representative scattering profiles for three classes of material, inorganics, polymers and metals. The top row [panels (*a*)–(*c*)] shows experimental data in absolute units of cm^−1^, while the bottom row [panels (*d*)–(*f*)] shows *I* multiplied by the typical thickness *t*_D_ (in centimetres) required for a device of the given material. For reference, the scattering from a Hellma cell (orange + symbols) is also included. The window materials (and thicknesses) considered are quartz (0.44 mm), fused silica (1.1 mm), soda lime glass (1.1 mm), borosilicate glass (0.14 mm), steel (0.1 mm) (Shin *et al.*, 2010 ▸), nickel (10 µm × 2) (Yoo *et al.*, 1982 ▸), aluminium (1 mm) and vanadium (1 mm) (Imae *et al.*, 2011 ▸), shown as a horizontal line.

Fig. 4 ▸ shows experimental scattering profiles for the various glass and silicon specimens, which are compared with selected polymers and metals. The data are presented in two ways: the scattering intensity in absolute units (cm^−1^) is shown in the top graphs. As mentioned above, this can be somewhat misleading, as we are interested in the scattering of the microdevice compared with that of a sample (confined to a given channel depth). The lower panels thus show the same data multiplied by the likely device thickness *t*_D_. The scattering signal for all glass and silicon specimens considered is weak compared with that of samples ‘compatible’ with microfluidic SANS, which should scatter above ∼1 cm^−1^ to enable reasonable acquisition times. Borosilicate exhibits higher scattering (in addition to lower device transmission) and is thus inferior to other glasses. By contrast, silicon is outstanding in this *q* range and thus even thick devices (of the order of centimetres) would be well suited for SANS. In general, neutron activation is not of concern, provided that the window thicknesses are kept to a minimum (tens to hundreds of micrometres).

#### 5.4.2. Polymers   

Polymer-based microdevices are generally inexpensive compared with glass and silicon devices and are amenable to rapid prototyping, *i.e.* device fabrication within hours of the initial design (Sollier *et al.*, 2011 ▸). Polymeric surface properties can be readily tailored by physical patterning or chemical modification (Becker & Gärtner, 2008 ▸), and a range of polymers are optically transparent which facilitates operation. However, polymers generally yield high incoherent signals (∼0.5–0.7 cm^−1^) owing to their high hydrogen content. The absolute scattering profiles of representative polymers are shown in Fig. 4 ▸(*b*), accompanied by their figures of merit *It*_D_ in the panel below, which are consistently above that of a Hellma cell. Polymers are generally classified into elastomers, thermosets and thermoplastics.

Elastomers, polymers with a rubber-like behaviour, exhibit high elasticity (low Young’s modulus) and high failure strain. By far the most commonly used elastomer in microfluidics is poly(dimethylsiloxane), PDMS (Whitesides, 2006 ▸). Devices are typically fabricated by replication against a mould (often an SU-8 master) and covalently sealed against glass or itself following surface oxidation (McDonald & Whitesides, 2002 ▸). Small feature sizes (∼1 µm, with sub-0.1 µm fidelity) can be generated with high accuracy in both planar and three-dimensional geometries. PDMS also offers good biocompatibility (Bélanger & Marois, 2001 ▸) and is permeable to certain gases, which can be advantageous, *e.g.* in biology. On the other hand, PDMS suffers from poor solvent compatibility as it is swollen by common organic solvents (Lee *et al.*, 2003 ▸). Furthermore, a conventional (∼1 cm thick) PDMS device would yield excessive neutron absorption and background scattering for SANS, with *It*_D_ ≃ 0.5 and *T* ≃ 0.6. Devising thin PDMS devices is certainly possible (Martin *et al.*, 2016 ▸) but, given its elastomeric nature, a thin membrane deforms under pressure, posing a problem in a scattering experiment, as the irradiated volume must be known for absolute intensity calibration.

Thermosets irreversibly cross link into solid networks, often by UV exposure, and include SU-8 (Lee & Sundararajan, 2010 ▸), the thiolene-based NOA-81 (Cabral *et al.*, 2004 ▸; Harrison *et al.*, 2004 ▸), acrylate formulations (*e.g.* Khoury *et al.*, 2002 ▸) and many others. Thermosets are often used as negative photoresists for master fabrication (open face) as well as for direct microdevice fabrication (closed face) *via* selective exposure with a photomask and development of the unconverted material. For example, NOA-81 has been used in both closed- and open-face lithography (Harrison *et al.*, 2004 ▸; Bartolo *et al.*, 2008 ▸), offering broad solvent compatibility (excluding chlorinated solvents; Cygan *et al.*, 2005 ▸) and minimum feature sizes of the order of 50 µm (Harrison *et al.*, 2004 ▸) to a few micrometres (Bartolo *et al.*, 2008 ▸). Thermoset polyester (TPE) or polyurethane methacrylate (PUMA) (Sollier *et al.*, 2011 ▸) yield devices with small features (down to ∼2 µm) capable of withstanding relatively large pressures (∼1 MPa or 10 bar).

Thermoplastic polymers, such as PS, PMMA and PC, soften above their glass transition temperature (*T*_g_) and microdevices can be fabricated by moulding under pressure, or shaped by other techniques including laser ablation. PMMA and *cyclo*-olefin-copolymer (COC) devices with wall thicknesses of 130–250 µm, and polyimide (Kapton) devices with a thickness of 75 µm, have been fabricated by hot embossing and laser ablation, respectively, and used in SAXS experiments (Metwally *et al.*, 2012 ▸; Marmiroli & Amenitsch, 2012 ▸; Dhouib *et al.*, 2009 ▸). A disadvantage of polymers such as PMMA is their incompatibility with a number of organic solvents, while Kapton on the other hand offers excellent solvent compatibility. Polyimide film devices have been fabricated by a range of procedures (Evans *et al.*, 2007 ▸; Trebbin *et al.*, 2013 ▸; Catalano *et al.*, 2014 ▸), yielding resolutions from ∼1 to 60 µm (Evans *et al.*, 2007 ▸) and varying solvent compatibility depending on the additional materials employed. An attractive feature of Kapton devices is the low thickness of the windows, down to ∼2 × 50 µm.

Highly structured polymers, such as semi-crystalline polymers (or ordered diblock copolymers), can scatter strongly in the high-*q* region and are therefore not recommended. Fig. 4 ▸(*b*) shows the scattering of five selected polymer systems: hydrogenated PS, PDMS, Kapton and (cross-linked) thiolene, in line with *I*(PMMA) ≃ 0.6 cm^−1^ and *I*(polyethylene) ≃ 0.62 cm^−1^ (Shibayama *et al.*, 2005 ▸) which are not shown. While it is possible to employ deuterated polymers to reduce incoherent scattering and absorption, their high(er) cost (∼100 USD g^−1^) and low availability may make this unfeasible. Deuteration yields an incoherent cross section ∼15 times lower that of hydrogenous polymers, resulting in potentially excellent window materials, in particular at high *q*, as shown by the d-PS profile. Hydrogenous polymeric devices therefore perform comparatively poorly for use in microfluidic SANS cells, unless thin-walled polymeric devices are used, provided that they are mechanically robust and enable robust inlet/outlet connections. Taking an illustrative channel depth of 500 µm, a device (top and bottom) wall thicknesses of ∼25 µm would be needed for the channels to scatter similarly to D_2_O (∼0.05 cm^−1^, a relatively weak scatterer). However, a number of ‘lab-on-a-foil’ polymer devices with windows down to 25 µm have been demonstrated in SAXS (Focke *et al.*, 2010 ▸; Tsao *et al.*, 2012 ▸). Alternatively, given the considerably lower background scattering of various glasses (approximately two orders of magnitude), it seems desirable to construct microdevices with glass windows and polymer matrices, thus combining ease of fabrication and low background, as discussed below.

#### 5.4.3. Metals and ceramics   

Metals offer high thermal and electric conductivity, and generally have high Young’s modulus, tensile strength and melting point. Gold, nickel and copper are the most commonly used metals in microfabrication (Lee & Sundararajan, 2010 ▸; Lang *et al.*, 2011 ▸), but microfluidic devices of several other metals, for example titanium, have also been reported (Lin *et al.*, 2012 ▸; Tong *et al.*, 2001 ▸; Chandrasekaran *et al.*, 2003 ▸; Kobayashi *et al.*, 2008 ▸; Zhang *et al.*, 2008 ▸). Fig. 4 ▸(*c*) shows the scattering profiles for nickel, aluminium, vanadium and steel, with the expected performance in a device shown by *It*_D_ in the row below. The ability to fabricate mechanically robust thin metal devices leads to generally good neutron cells, in particular in the range *q* 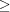 0.05 Å^−1^. For example, nickel devices with 10 µm thick walls (Lang *et al.*, 2011 ▸) are capable of withstanding pressures up to ∼6 bar (90 psi). Aluminium is the weakest scatterer of the metals tested and, even with a 1 mm reference thickness, it compares favourably with quartz or fused silica in the high-*q* region (*q* 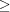 0.1 Å^−1^), although its quality decreases at lower *q* values, reaching the value of D_2_O at *q* ≃ 0.02 Å^−1^. However, we note the limited chemical stability of aluminium, and its susceptibility to solutions of varying salt and pH can impact the surface roughness and composition of the microchannels.

Ceramics are inorganic, hard insulating materials with high melting temperatures and excellent chemical resistance, which have been employed recently for microfabrication (Golonka *et al.*, 2006 ▸; Gómez-de Pedro *et al.*, 2010 ▸; Schindler & Roosen, 2009 ▸). For instance, microdevices based on Dupont 951 green tape (http://www.dupont.com; Jones *et al.*, 2000 ▸; Rodriguez *et al.*, 2000 ▸) have been fabricated by a variety of methods, with wall thicknesses in the 50–250 µm range, with (estimated) high transmission (∼0.98 mm^−1^) and low incoherent and background scattering (*I* ≃ 0.0002–0.005 cm^−1^).

Other materials used in microfabrication, such as paper or hydrogels, are not considered here since these do not exhibit high flow resistance and are expected to yield high scattering background.

## 6. Microdevice fabrication   

We consider several microfabrication approaches which appear particularly well suited for microfluidic SANS. Polymeric (and hybrid) devices are amenable to rapid prototyping in most university laboratories, are inexpensive, and enable iterations and refinements, which are particularly important for flow-SANS experiments. By contrast, microfabrication in glass or metal, for instance, generally offers more durable devices, able to sustain higher pressures and attractive for repeated or prolonged use, such as in phase mapping. Key features of selected materials and approaches are summarized in Table 3 ▸. Amongst these, we highlight three methods that appear particularly well suited to microfluidic SANS.

### 6.1. Method 1: closed-face photolithography and reinforcement   

**Figure 5:**
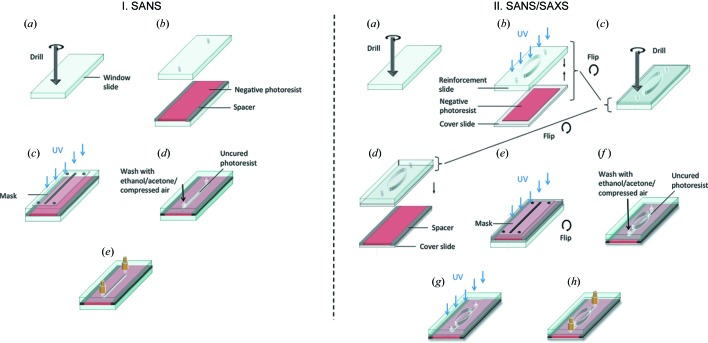
Schematic of microfabrication procedure (method 1) based on a closed-face lithography approach (Cabral *et al.*, 2004 ▸; Harrison *et al.*, 2004 ▸) and employed in flow-SANS (Lopez *et al.*, 2015 ▸). Photopolymerization is carried out between two ∼1 mm (boron-free) glass sheets (left). If a thin (∼100 µm) slide is used instead, for instance to enable SAXS measurements as well as SANS, the sandwich is reinforced with a thick glass plate to ensure mechanical integrity (right). The channel geometry is imposed by a photomask and development (see text for details).

Given the low background scattering of (boron-free) glass and quartz and the ease of fabrication of photopolymerization, an attractive fabrication approach consists of so-called ‘closed-face’ lithography, illustrated in Fig. 5 ▸. The method consists of the photopolymerization of a UV-curable resin between UV-transparent plates using a photomask, and then the displacement of the unpolymerized liquid to yield the microchannels. The window materials must be transparent to the radiation used for curing (typically UV), and the photoresist must adhere strongly to the window material to withstand pressure. Quartz, fused silica, crown glass and soda lime glass offer good optical and neutron properties and are thus suitable windows. Handling, drilling and port connection require the use of either relatively thick (∼1–1.2 mm) or reinforced thin cover slides (∼100–150 µm), as described below.

The detailed procedure is illustrated in Fig. 5 ▸. Panel I (left) shows the simplest procedure, reported earlier by Harrison *et al.* (2004 ▸) and here employing ∼1 mm boron-free glass (*e.g.* B270, Schott AG) or quartz plates. Panel II depicts a longer procedure, yielding devices compatible with both SANS and SAXS. The procedure is as follows. (*a*) Small holes are drilled in a thick (1 mm) glass slide which serve as inlet(s) and outlet, and a large hole/window is drilled around the region for observation with neutrons. We find that drilling the inlet and outlet holes before the large central one significantly reduces breakages while drilling. This slide will act as a ‘reinforcement’ for the thinner glass slides (*e.g. t* = 140 µm borosilicate glass slides). A conventional high-speed drill with a diamond drill bit can be employed. (*b*) A few drops of thiolene (NOA81) are cast onto a thin cover slide and the reinforcement slide is sealed under UV light, any excess adhesive being removed with a razor blade. (*c*) Holes are drilled through the thin cover slide on the inlet(s) and outlet positions; the thin and thick slides are then bonded together prior to device patterning. (*d*) To define a precise channel depth, spacers are placed on top of another thin cover slide, thiolene is poured on top and the reinforced slide sandwiches the thiolene resist layer. (*e*) The device is flipped, a negative photomask (*i.e.* with microchannels in black) is placed over the assembly and the whole is exposed to a prescribed UV dose (Cabral *et al.*, 2004 ▸) to yield the desired channel depth (*e.g.* 5 min at 0.14 J cm^−2^ for a 540 µm deep channel). (*f*) The liquid thiolene that remains under the masked sections is flushed out using ethanol, acetone and compressed air. (*g*) The device is post cured by exposure to high-intensity UV for at least 30 min. (*h*) Nanoports (or equivalent) are sealed to the device using fast-curing Araldite glue, and the device is ready for operation. The entire process takes approximately 1 h, yielding devices suitable for SANS (Lopez *et al.*, 2015 ▸) and SAXS (Poulos *et al.*, 2016 ▸).

The minimum feature size obtainable with this method is ∼50–100 µm, depending on the aspect ratio and process parameters (Harrison *et al.*, 2004 ▸). Open-face replication methods (Bartolo *et al.*, 2008 ▸) or the use of a range of other monomer/polymer resists (Vitale *et al.*, 2015 ▸) can further reduce these to a few micrometres. Multi-level patterning of the microchannels is possible by controlling the frontal photopolymerization process of the resist (Cabral *et al.*, 2004 ▸; Cabral & Douglas, 2005 ▸).

Overall, this simple lithographic technique yields solvent-resistant non-deformable microdevices, with transmissions up to ∼98% with quartz windows (or similar with soda lime, crown glass or fused silica). Borosilicate glass has a similarly low background but much lower transmission, and suitable devices must therefore have thin walls (∼2 × 140 µm for 80% transmission).

### 6.2. Method 2: hybrid PDMS–thiolene devices   

**Figure 6:**
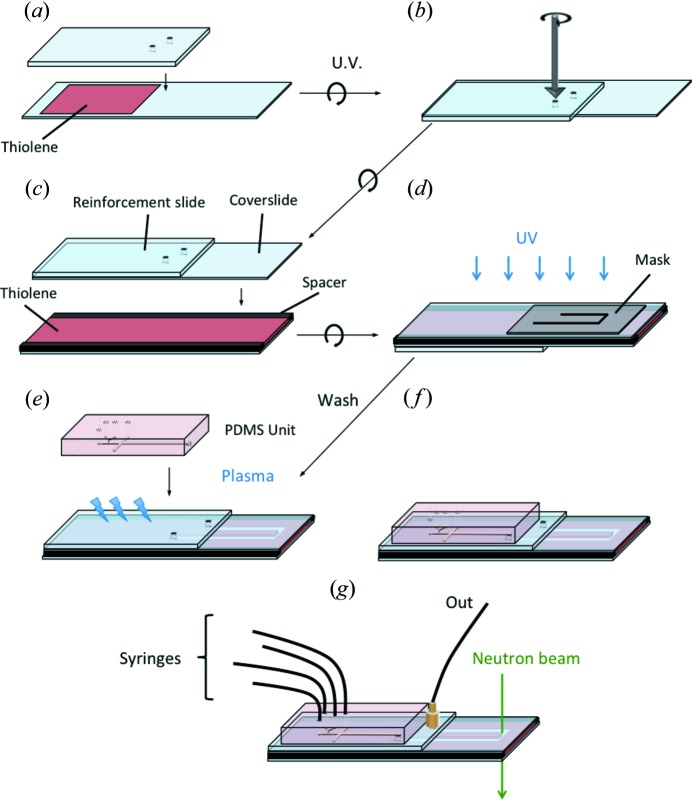
Schematic of PDMS–thiolene hybrid microdevice fabrication (method 2), combining method 1 with PDMS fabrication and sealing to enable higher density and smaller-scale patterning, prior to measurement.

While the fabrication of PDMS devices by SU-8 mould replication is well established (Duffy *et al.*, 1998 ▸) and enables small scale and densely packed microchannels, the high neutron background and channel deformation under flow of thin PDMS membranes is undesirable. Reinforcement approaches are possible (Martin *et al.*, 2016 ▸), but a combination of PDMS and photolithography, illustrated in Fig. 6 ▸, appears more attractive. The procedure follows the steps of the previous method until step (*d*). After the channels have been developed, a replicated PDMS slab (moulded on an SU-8 master following the usual procedures; McDonald & Whitesides, 2002 ▸) is oxidized, aligned with the glass ports and irreversibly sealed using a plasma oven, as shown in Figs. 6 ▸(*e*) and 6 ▸(*f*). Finally, in step (*g*) tubing is connected to the PDMS module onto pre-cored ports, and an outlet connector is attached through the reinforcement slide.

The small feature sizes available with PDMS can then be used for fast mixing, formulation or flow processing of complex fluids, allowing the sample to be analysed through a high-quality observation window. With this method it is not possible to carry out truly *in situ* SANS under flow, as the fluid has to be transported from the PDMS section into the window, which typically has a dead volume of ∼1 µl.

### 6.3. Method 3: glass devices (etching and bonding, and laser etching)   

**Figure 7:**
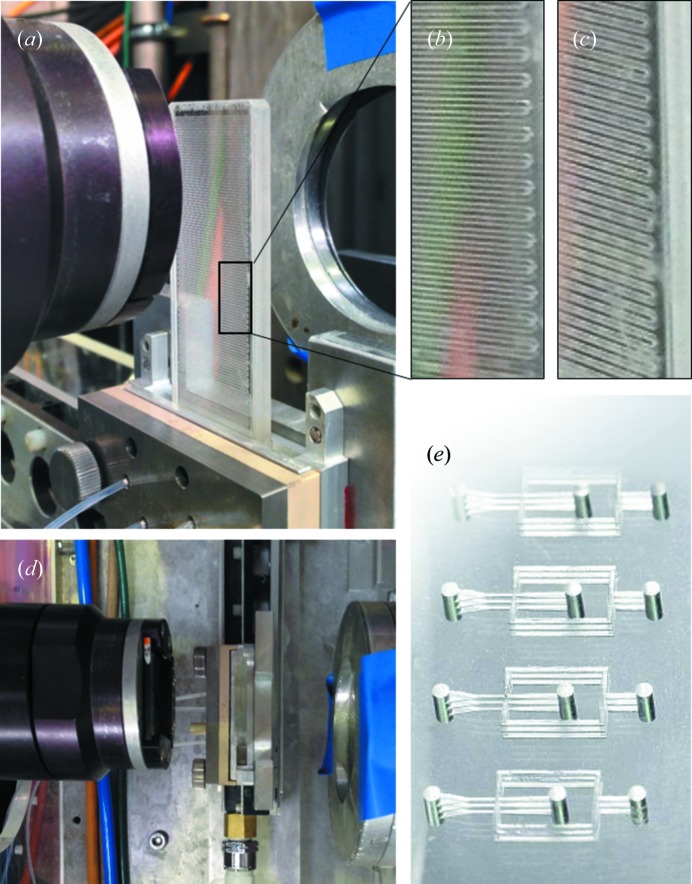
(*a*) Crown glass chip (Dolomite microreactor) installed on the SANS beamline D22 at ILL, operating in (*b*) continuous and (*c*) droplet modes. (*d*) A top view of the setup, showing the beam and detector tubes. (*e*) A fused silica microchip fabricated by selective laser etching [reproduced with permission from LightFab (2018 ▸), copyright (2018) LightFab GmbH, Aachen, Germany].

All-glass devices can offer superior performance compared with methods 1 and 2 in terms of durability and chemical and pressure resistance, which may warrant the higher material and processing costs required. While glass micromachining, etching and bonding (Iliescu *et al.*, 2012 ▸) generally involve specialized equipment, a number of commercial suppliers offer a range of devices that seem eminently compatible with microfluidic SANS, provided that a suitable grade of (boron-free) glass is employed. Typically, channels are patterned with use of a masking layer, followed by wet etching with hydrofluoric acid, or dry etching (*e.g.* with deep reactive-ion etching, DRIE) for high-aspect-ratio channels. Access ports for inlets or outlets can also be wet etched, as well as drilled or sand blasted. Alternative processes couple laser ablation with chemical etching (laser-induced deep etching; http://www.lpkf.com/applications/lide-technology/). Finally, the patterned surface is bonded by direct bonding or fusion bonding at high (∼923 K) temperatures or with an adhesive, before the fluidic ports are connected. Commercial suppliers often make available robust mounting platforms with push-fit tubing or ferrule connectors for additional robustness (*e.g.* against leaks or detachment). Typically, these devices can withstand pressures in excess of 100 bar (and generally limited by the connectors). While borosilicate glass is generally unsuitable because of its high absorption (see Table S1 in the supporting information), a common and inexpensive crown glass (*e.g.* B270 ultra white glass, Schott AG) with a low boron content is well suited, as demonstrated in previous contrast-variation and phase-mapping experiments (Adamo *et al.*, 2017 ▸; Adamo *et al.*, 2018 ▸) employing a Dolomite 4 mm thick microreactor chip (illustrated in Figs. 7 ▸ *a*–7 ▸ *d*).

Selective laser-induced etching methods [see, for instance, LightFab (2018 ▸) and Meineke *et al.* (2016 ▸)] are emerging as subtractive three-dimensional printing approaches capable of generating buried microchannels within glass (fused silica and quartz), avoiding the need for glueing or bonding separate sections (Figs. 7 ▸ *e*). These typically employ ultrafast pulsed lasers and focusing optics or two-photon processes to pattern directly inside the glass matrix (or, equivalently, within polymeric matrices). The spatial resolution can be as high as 1–2 µm and the high optical transparency, chemical, thermal and pressure resistance, and neutron transmission make these approaches particularly suitable for SANS device fabrication.

### 6.4. A comparison of existing and emerging microfabrication methods   

Table 3 ▸ compiles representative microfabrication techniques, along with relevant properties characterizing the SANS quality of the devices and key practical considerations. Evidently, a number of materials and approaches appear suitable for microfluidic SANS, with hydrogenated polymers faring comparatively poorly, especially elastomers such as PDMS. Boron-free glasses (method 3) and metals emerge as good candidates, provided that neutron activation can be kept low, either by material composition or by thin channel walls. While the associated fabrication methods generally involve longer manufacturing times and are more costly than photolithography methods, these generally provide superior longevity, spatial resolution, pressure resistance and, often, SANS quality (in terms of transmission and background scattering).

Methods 1 and 2 proposed here aim to combine the high neutron quality of glass or quartz with the versatility and speed offered by polymer microfabrication. These yield high-quality neutron cells that can be manufactured within hours of initial design, albeit suffering from limited resolution (partially overcome by method 2). Their low fabrication cost (∼10 USD) and time (of the order of hours) mean that they are effectively disposable and device cleaning might thus not be needed (compared with ∼1000 USD for a commercial glass device, and of the order of weeks for custom manufacturing). Devices fabricated by method 3 using thin (*e.g.* borosilicate) slides are also compatible with SAXS (which commonly uses thin-walled ∼50 µm glass capillaries as liquid sample cells).

The advent of facile three-dimensional printing and additive manufacturing of polymers, glasses, metals and ceramics will undoubtedly continue to broaden versatility, lower the cost and increase the speed of rapid prototyping (Bhattacharjee *et al.*, 2016 ▸), and are expected to further enable microfluidic SANS. High-precision methods of laser micromachining and etching, which can provide exceptional dimensional control for microdevice fabrication, are becoming increasingly accessible and are well placed to meet SANS requirements.

The varied nature of microfluidic SANS experiments, ranging from flow-SANS and process kinetics to systematic phase-space exploration, will probably require a combination of rapid prototyping and fast iteration, alongside precision manufacturing approaches.

## 7. Conclusions   

We have reviewed materials and approaches for the fabrication of microfluidic devices compatible with SANS. A few closed-face photopolymerization methods between quartz (or boron-free glass) plates emerge as effective rapid prototyping approaches, requiring facilities available in most chemical laboratories, and enable rapid iterations that are advantageous for some SANS experiments (*e.g.* flow-SANS). A number of glass etching and bonding techniques, as well as buried laser etching techniques, provide durable solvent-compatible optically transparent high-pressure microdevices that are particularly well suited for microfluidic SANS, in particular for applications requiring prolonged or repeated use (*e.g.* phase mapping). The combination of both approaches, in addition to continued advances in the three-dimensional printing of polymers and glasses, is likely to meet most requirements for microfluidic SANS.

**Figure 8:**
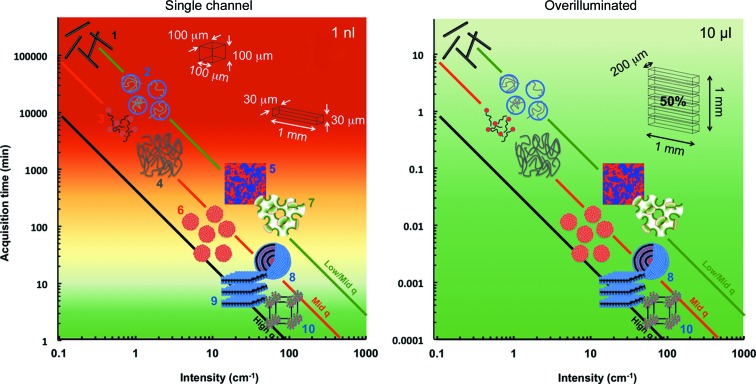
System compatibility with microfluidic SANS. Acquisition times are plotted for representative systems based on their characteristic scattering intensity (cm^−1^), sample volumes of 1 nl and 10 µl, and the *q*-range/configuration required [defined as high (0.05–0.5 Å^−1^), mid (0.01–0.2 Å^−1^) and mid/low (0.005–0.05 Å^−1^) *q*]. Adapted and expanded from Lopez *et al.* (2015 ▸) under a Creative Commons Attribution 4.0 International License, https://creativecommons.org/licenses/by/4.0/.

Inevitably, microfluidic approaches reduce the SANS sampling volume, whether by interrogating single microchannels or in flow mapping with a small beam, or by overilluminating several channels, as illustrated in Fig. 1 ▸. We therefore conclude with an assessment of the current feasibility of such experiments, based on acquisition times estimated for representative soft-matter systems, *q* configurations and illuminated volumes, illustrated in Fig. 8 ▸. For illustration, we consider (i) a sample volume of 1 nl, corresponding to a beam of diameter ∼100 µm and a channel depth of 100 µm, representative of a single-channel configuration [see *e.g.* Lopez *et al.* (2015 ▸) for a volume ∼10 nl], and (ii) a sample volume of 10 µl, comparable to the overilluminated channels relevant for phase mapping (Adamo *et al.*, 2017 ▸, 2018 ▸). We divide the *q* range accessible in a typical SANS instrument into three representative windows: high *q* (0.05–0.5 Å^−1^), mid *q* (0.01–0.2 Å^−1^) and low *q* (0.005–0.05 Å^−1^). Data acquisition times generally increase towards lower *q* ranges owing to a combination of a smaller solid angle and/or lower flux for long-wavelength neutrons, making high- and mid-*q* configurations particularly suitable for microfluidic SANS. Considering the inverse proportionality between acquisition time and both the absolute scattering intensity and (illuminated) sample volume, the experimental feasibility is indicated by colour in Fig. 8 ▸, according to the time required per measurement. The boundary between ‘feasible’ (green) and ‘unfeasible’ (red) is tentatively set at acquisition times of ∼1–10 min per measurement. For the single-channel setup with ∼100 µm beams, only concentrated ordered systems are practical in microfluidic SANS with current neutron fluxes. However, ∼1 s acquisitions are often sufficient to determine structural features like a peak position and order parameter, as demonstrated in Fig. 1 ▸(*f*). For channel overillumination, and beams of the order of centimetres, a large range of systems are compatible with microfluidic phase-mapping SANS. As a rule of thumb, the channel matrix (*i.e.* the spacing between channels) is 20–50% of the channel width, channel depths are sub-millimetre and counting times need to be adjusted accordingly with respect to the bulk sample. Further, operational considerations of the required mixing times and flow dispersion, relevant for the precise formulation of mixtures, must be carefully considered (Adamo *et al.*, 2017 ▸).

Overall, microfluidics offers unprecedented opportunities to expedite traditional SANS experiments (*e.g.* phase-space exploration) or to unlock the molecular or mesoscopic underpinnings of complex fluid rheology and processing, and the mechanisms and kinetics of phase and conformation transition kinetics, amongst others. This review provides clear recommendations for choice of materials and fabrication methods for microfluidic SANS.

## 8. Related literature   

For further literature related to the supporting information, see Brûlet *et al.* (2007 ▸), Dolník *et al.* (2000 ▸), Glinka (2011 ▸), Hammouda (2008 ▸), James & Doremus (2008 ▸), Lide (2003 ▸), NIST (2018 ▸), Osman *et al.* (2015 ▸), Prince *et al.* (1999 ▸), Root *et al.* (1989 ▸) and Sears (1992 ▸).

## Supplementary Material

Theoretical background and additional tables and figures. DOI: 10.1107/S1600576718007264/aj5307sup1.pdf

## Figures and Tables

**Table 1:** Representative flow cells employed with SANS

Method	Flow type	Fabrication material	References
Flow-SANS	Poiseuille	Quartz	Cloke *et al.* (1996 ▸)
	Contraction–expansion	Aluminium, sapphire	Bent, Hutchings *et al.* (2003 ▸), Bent, Richards & Gough (2003 ▸), Lutz-Bueno *et al.* (2015 ▸)
	Opposing-jet, cross-flow	Quartz	Penfold *et al.* (2006 ▸), Qazi *et al.* (2011 ▸)
Rheo-SANS	Couette	Quartz, aluminium, titanium	Gurnon *et al.* (2014 ▸), Takeda *et al.* (2011 ▸), Liberatore *et al.* (2006 ▸), Gentile *et al.* (2011 ▸), Egres *et al.* (2006 ▸), Yearley *et al.* (2010 ▸), Lindner & Oberthür (1984 ▸), Cummins *et al.* (1990 ▸)
	Plate–plate	Quartz, aluminium	Kalus *et al.* (1990 ▸), Tepe *et al.* (1995 ▸), Sharma *et al.* (2010 ▸)
	Slit	Quartz	Weston *et al.* (2018 ▸)
Stopped-flow and other setups	Stopped flow	Quartz	Reviewed by Grillo (2009 ▸)
	Size-exclusion chromatography	Quartz	Jordan *et al.* (2016 ▸)
	Flow-through cell	Quartz	Metze *et al.* (2017 ▸), Hayward *et al.* (2018) ▸

**Table 2:** Decay times to 0.1 nCi radioactivity for different materials, masses and exposure times Values are calculated for a reference flux of 10^8^ neutrons cm^−2^ s^−1^, employing the NIST neutron activation calculator (NIST, 2017 ▸).

	Mass (g)					
	1		10^−3^		5 × 10^−5^	
	Exposure time (h)					
	1	10	1	10	1	10
Material	Decay time					
Copper	8 d	10 d	3 d	4 d	14 h	2.2 d
Nickel	1 d	18 y	–	5 h	–	–
Cobalt	38 y	56 y	2 h	3 y	2 h	2 h
Titanium	1 h	1 h	10 min	10 min	–	–
Zinc	2 y	5 y	4 h	9 h	–	1 h
Iron	1 y	10 y	–	–	–	–
Aluminium	1 h	1 h	–	–	–	–
Palladium	2 months	4 months	2 d	4 d	–	2 d
Chromium	7 months	1 y	–	1 month	–	–
SiO_2_	20 h	1 d	–	–	–	–
Si	23 h	1 d	–	1 h	–	–
Borosilicate	20 h	1 d	–	–	–	–
h-PMMA/d-PMMA	–	–	–	–	–	–

**Table:** A reference for the fabrication procedure is provided in the top row. Additional references for specific values are included in the relevant boxes.

	Silicon^*a*^	Glass/quartz^*a*^	Aluminium/glass^*b*^	Nickel^*c*^	Nickel/silicon^*d*^	NOA-81 closed lithography^*e*^
Lateral resolution (µm)	1^*a*^	1^*a*^	100^*f*,*g*^	20	20^*d*^	50–100^*e*^
*t* (mm)	^*g*^	^*g*^	5^*g*^	0.02	0.025	0.28–1^*h*^
*I*_back_ (cm^−1^)	0.0007	0.004	0.003	0.03	0.03	0.004
*I*_back_ *t*	0.00035	0.002	0.003	0.00006	0.00008	0.0004
*I*(*q* = 0.01 Å^−1^)*t*	0.00035	0.002	0.5	0.002	0.002	0.0004
Transmission	1	0.9–0.95	0.8	1	1	0.98/0.78
Cost	−	−	+	+	−	++
Rapid prototyping	−	−	−	−	=	++
Pressure resistance (bar)	+	100	+	6	+	5^*i*^
Solvent compatibility	++^*j*^	++^*j*^	+	++	++	+^*e*^
Equipment requirements	+	+	−	+	−	++
Notes	Optically opaque	Commercially available	Commercially available	Optically opaque	Optically opaque	Compatible with SAXS^*k*^

**Table:** 

	NOA-81 stickers^*l*^	Kapton/PDMS^*m*^	Kapton laser ablation^*n*^	h-Polymer hot embossing^*o*^	d-Polymer hot embossing^*o*^	PDMS^*p*^
Lateral resolution (µm)	1–10	1^*q*^	10	1^*o*^	1^*o*^	1–10^*r*^
*t* (mm)	0.4	0.07	0.15^*n*^	0.2–0.3^*s*^	0.2–0.3^*s*^	1–10^*s*,*t*^
*I*_back_ (cm^−1^)	0.53	0.6^*u*^	0.6^*u*^	0.5–0.7	0.03	0.5
*I*_back_ *t*	0.02	0.004	0.009	0.015	0.001	0.06
*I*(*q* = 0.01 Å^−1^)*t*	0.02	0.03	0.06	0.015	0.06	0.09
Transmission	0.8	0.96	0.97	0.5–0.7	0.97	0.6
Cost	++	+	++	++	−	+
Rapid prototyping	+	+	−	−	−	+
Pressure resistance (bar)	5^*i*^	−	+	10^*t*^	10^*t*^	3^*v*^
Solvent compatibility	+^*e*^	−	++	−	−	−^*w*^
Equipment requirements	+	+	−	−	−	+
Notes	Compatible with SAXS and light scattering^*x*^	Compatible with SAXS; PDMS can be replaced by metal, resolution ∼100 µm	Compatible with SAXS	Compatible with SAXS	Compatible with SAXS	Deforms under pressure

(*a*) See Iliescu *et al.* (2012 ▸) and Lee & Sundararajan (2010 ▸), and references therein, for a variety of methods available to fabricate devices of this kind. (*b*) Lin *et al.* (2012 ▸). (*c*) Lang *et al.* (2011 ▸). (*d*) Papautsky *et al.* (1998 ▸). Foil thickness range 15–50 µm, with an average value of 35 µm selected here. Additional scattering from silicon and a thin gold layer is not considered as it is negligible compared with that from nickel. (*e*) Cabral *et al.* (2004 ▸). (*f*) Smaller features are possible (Adams *et al.*, 2001 ▸). (*g*) The device thickness varies depending on the fabrication method but is generally unimportant owing to weak scattering and absorption; a reference value of 5 mm was chosen. (*h*) See §6. (*i*) Sollier *et al.* (2011 ▸). (*j*) Becker & Gärtner (2008 ▸). (*k*) Poulos *et al.* (2016 ▸). (*l*) Microfluidic stickers fabricated only with thiolene (Brennich *et al.*, 2011 ▸), as considered here, or glass/thiolene/glass devices (Bartolo *et al.*, 2008 ▸) (for which rows ‘*t*’ to ‘Transmission’ would take the values of the closed lithography case). (*m*) Evans *et al.* (2007 ▸). Replacing PDMS with metal (Koester & Pfohl, 2012 ▸) increases the solvent compatibility ‘++’ and the lateral resolution is limited to ∼60 µm. (*n*) Barrett *et al.* (2006 ▸). (*o*) Becker & Dietz (1998 ▸), Heckele & Schomburg (2003 ▸), Goral *et al.* (2010 ▸) and Becker & Heim (2000 ▸). (*p*) Martin *et al.* (2016 ▸). (*q*) Dootz *et al.* (2007 ▸) and McDonald & Whitesides (2002 ▸). (*r*) McDonald & Whitesides (2002 ▸). (*s*) Metwally *et al.* (2012 ▸) and Dhouib *et al.* (2009 ▸). (*t*) See Sollier *et al.* (2011 ▸) for TPE and PUMA. (*u*) The maximum value at the peak is taken, rather than the background intensity. (*v*) Deforms under high pressures, see Bartolo *et al.* (2008 ▸). (*w*) Lee *et al.* (2003 ▸). (*x*) Norman *et al.* (2006 ▸).

## References

[bb2] Adamo, M., Poulos, A. S., Lopez, C. G., Martel, A., Porcar, L. & Cabral, J. T. (2018). *Soft Matter*, **14**, 1759–1770. 10.1039/c7sm02433a 29355865

[bb1] Adamo, M., Poulos, A., Miller, R. M., Lopez, C. G., Martel, A., Porcar, L. & Cabral, J. T. (2017). *Lab Chip*, **17**, 1559–1569. 10.1039/c7lc00179g 28379253

[bb201] Adams, D. P., Vasile, M. J., Benavides, G. & Campbell, A. N. (2001). *Precision Eng.* **25**, 107–113.

[bb3] Bailey, I. (2003). *Z. Kristallogr. Cryst. Mater.* **218**, 84–95.

[bb4] Barker, J. G. & Mildner, D. F. R. (2015). *J. Appl. Cryst.* **48**, 1055–1071. 10.1107/S1600576715010729 PMC4520287 26306088

[bb202] Barrett, R., Faucon, M., Lopez, J., Cristobal, G., Destremaut, F., Dodge, A., Guillot, P., Laval, P., Masselon, C. & Salmon, J.-B. (2006).* Lab Chip,* **6**, 494–499. 10.1039/b517055a 16572211

[bb5] Bartolo, D. & Aarts, D. G. (2012). *Soft Matter*, **8**, 10530–10535.

[bb6] Bartolo, D., Degré, G., Nghe, P. & Studer, V. (2008). *Lab Chip*, **8**, 274–279. 10.1039/b712368j 18231666

[bb203] Becker, H. & Dietz, W. (1998). *Proc. SPIE*, **3515**, 177–181.

[bb7] Becker, H. & Gärtner, C. (2008). *Anal. Bioanal. Chem.* **390**, 89–111. 10.1007/s00216-007-1692-2 17989961

[bb206] Becker, H. & Heim, U. (2000). *Sensors Actuators A Phys.* **83**, 130–135

[bb8] Bélanger, M.-C. & Marois, Y. (2001). *J. Biomed. Mater. Res.* **58**, 467–477. 10.1002/jbm.1043 11505420

[bb9] Bent, J., Hutchings, L., Richards, R., Gough, T., Spares, R., Coates, P. D., Grillo, I., Harlen, O., Read, D., Graham, R., Likhtman, A., Groves, D. J., Nicholson, T. M. & McLeish, T. C. B. (2003). *Science*, **301**, 1691–1695. 10.1126/science.1086952 14500974

[bb10] Bent, J. F., Richards, R. W. & Gough, T. D. (2003). *Rev. Sci. Instrum.* **74**, 4052–4057.

[bb11] Beuvier, T., Panduro, E. A. C., Kwaśniewski, P., Marre, S., Lecoutre, C., Garrabos, Y., Aymonier, C., Calvignac, B. & Gibaud, A. (2015). *Lab Chip*, **15**, 2002–2008. 10.1039/c5lc00115c 25792250

[bb12] Bhattacharjee, N., Urrios, A., Kang, S. & Folch, A. (2016). *Lab Chip*, **16**, 1720–1742. 10.1039/c6lc00163g PMC4862901 27101171

[bb13] Brennich, M. E., Nolting, J.-F., Dammann, C., Nöding, B., Bauch, S., Herrmann, H., Pfohl, T. & Köster, S. (2011). *Lab Chip*, **11**, 708–716. 10.1039/c0lc00319k 21212871

[bb14] Bretagne, A., Cotot, F., Arnaud-Roux, M., Sztucki, M., Cabane, B. & Galey, J.-B. (2017). *Soft Matter*, **13**, 3812–3821. 10.1039/c6sm02510b 28485735

[bb104] Brûlet, A., Lairez, D., Lapp, A. & Cotton, J.-P. (2007). *J. Appl. Cryst.* **40**, 165–177.

[bb15] Cabral, J. T. & Douglas, J. F. (2005). *Polymer*, **46**, 4230–4241.

[bb16] Cabral, J. T., Hudson, S. D., Harrison, C. & Douglas, J. F. (2004). *Langmuir*, **20**, 10020–10029. 10.1021/la049501e 15518489

[bb17] Catalano, R., Perozziello, G., Simone, G., Candeloro, P., Gentile, F., Coluccio, M. L., Pardeo, F., Burghammer, M., Cuda, G., Riekel, C. & Di Fabrizio, E. (2014). *Microelectron. Eng.* **124**, 13–16.

[bb18] Chandrasekaran, S., Brazzle, J. D. & Frazier, A. B. (2003). *J. Microelectromech. Syst.* **12**, 281–288.

[bb19] Cloke, V. M., Higgins, J. S., Phoon, C. L., Richardson, S. M., King, S. M., Done, R. & Cooper, T. E. (1996). *Rev. Sci. Instrum.* **67**, 3158–3163.

[bb20] Cummins, P., Staples, E., Millen, B. & Penfold, J. (1990). *Meas. Sci. Technol.* **1**, 179–183.

[bb21] Cygan, Z. T., Cabral, J. T., Beers, K. L. & Amis, E. J. (2005). *Langmuir*, **21**, 3629–3634. 10.1021/la0471137 15807612

[bb22] Dhouib, K., Khan Malek, C., Pfleging, W., Gauthier-Manuel, B., Duffait, R., Thuillier, G., Ferrigno, R., Jacquamet, L., Ohana, J., Ferrer, J.-L., Théobald-Dietrich, A., Giegé, R., Lorber, B. & Sauter, C. (2009*a*). *Lab Chip*, **9**, 1412–1421. 10.1039/b819362b 19417908

[bb23] Dittrich, P. S., Tachikawa, K. & Manz, A. (2006). *Anal. Chem.* **78**, 3887–3908. 10.1021/ac0605602 16771530

[bb105] Dolník, V., Liu, S. & Jovanovich, S. (2000). *Electrophoresis*, **21**, 41–54. 10.1002/(SICI)1522-2683(20000101)21:1<41::AID-ELPS41>3.0.CO;2-7 10634469

[bb207] Dootz, R., Evans, H., Köster, S. & Pfohl, T. (2007). *Small*, **3**, 96–100. 10.1002/smll.200600288 17294477

[bb24] Duffy, D. C., McDonald, J. C., Schueller, O. J. & Whitesides, G. M. (1998). *Anal. Chem.* **70**, 4974–4984. 10.1021/ac980656z 21644679

[bb26] Eberle, A. P. & Porcar, L. (2012). *Curr. Opin. Colloid Interface Sci.* **17**, 33–43.

[bb27] Egres, R. G., Nettesheim, F. & Wagner, N. J. (2006). *J. Rheol.* **50**, 685–709.

[bb28] Evans, H., Dootz, R., Köster, S., Struth, B. & Pfohl, T. (2007). *Bull. Pol. Acad. Sci. Tech. Sci.* **55**, 217–227.

[bb29] Focke, M., Kosse, D., Müller, C., Reinecke, H., Zengerle, R. & von Stetten, F. (2010). *Lab Chip*, **10**, 1365–1386. 10.1039/c001195a 20369211

[bb30] Gentile, L., Rossi, C. O., Olsson, U. & Ranieri, G. A. (2011). *Langmuir*, **27**, 2088–2092. 10.1021/la1046047 21261313

[bb31] Ghazal, A., Lafleur, J. P., Mortensen, K., Kutter, J. P., Arleth, L. & Jensen, G. V. (2016). *Lab Chip*, **16**, 4263–4295. 10.1039/c6lc00888g 27731448

[bb106] Glinka, C. J. (2011). *J. Appl. Cryst.* **44**, 618–624.

[bb32] Golonka, L. J., Zawada, T., Radojewski, J., Roguszczak, H. & Stefanow, M. (2006). *Int. J. Appl. Ceram. Technol.* **3**, 150–156.

[bb68] Gómez-de Pedro, S., Puyol, M. & Alonso-Chamarro, J. (2010). *Nanotechnology*, **21**, 415603. 10.1088/0957-4484/21/41/415603 20844325

[bb205] Goral, V. N., Hsieh, Y.-C., Petzold, O. N., Faris, R. A. & Yuen, P. K. (2010). *J. Micromech. Microeng.* **21**, 017002.

[bb33] Graceffa, R., Nobrega, R. P., Barrea, R. A., Kathuria, S. V., Chakravarthy, S., Bilsel, O. & Irving, T. C. (2013). *J. Synchrotron Rad.* **20**, 820–825. 10.1107/S0909049513021833 PMC3795536 24121320

[bb34] Greaves, E. D. & Manz, A. (2005). *Lab Chip*, **5**, 382–391. 10.1039/b415836a 15791335

[bb35] Grillo, I. (2009). *Curr. Opin. Colloid Interface Sci.* **14**, 402–408.

[bb36] Gurnon, A. K., Godfrin, P. D., Wagner, N. J., Eberle, A. P., Butler, P. & Porcar, L. (2014). *J. Visualized Exp.* **84**, 51068. 10.3791/51068 PMC4116790 24561395

[bb107] Hammouda, B. (2008). *Probing Nanoscale Structures: the SANS Toolbox*. NIST Center for Neutron Research, Gaithersburg, Maryland, USA. https://www.ncnr.nist.gov/staff/hammouda/the_sans_toolbox.pdf

[bb37] Harrison, C., Cabral, J. T., Stafford, C. M., Karim, A. & Amis, E. J. (2004). *J. Micromech. Microeng.* **14**, 153–158.

[bb115] Hayward, D. W., Chiappisi, L., Prévost, S., Schweins, R. & Gradzielski, M. (2018). *Sci. Rep.* **8**, 7299. 10.1038/s41598-018-24718-z PMC5940810 29740024

[bb204] Heckele, M. & Schomburg, W. (2003). *J. Micromech. Microeng.* **14**, R1.

[bb38] Hudson, S. D., Phelan, F. R. Jr, Handler, M. D., Cabral, J. T., Migler, K. B. & Amis, E. J. (2004). *Appl. Phys. Lett.* **85**, 335–337.

[bb39] Iliescu, C., Taylor, H., Avram, M., Miao, J. & Franssila, S. (2012). *Biomicrofluidics*, **6**, 016505. 10.1063/1.3689939 PMC3365353 22662101

[bb40] Imae, T., Kanaya, T., Furusaka, M. & Torikai, N. (2011). *Neutrons in Soft Matter.* Chichester: John Wiley and Sons.

[bb108] James, F. & Doremus, R. (2008). *Ceramic and Glass Materials: Structure, Properties and Processing*. London: Springer.

[bb41] Jones, W. K., Liu, Y., Larsen, B., Wang, P. & Zampino, M. (2000). *Int. J. Microcircuits Electron. Packag.* **23**, 469–473.

[bb42] Jordan, A., Jacques, M., Merrick, C., Devos, J., Forsyth, V. T., Porcar, L. & Martel, A. (2016). *J. Appl. Cryst.* **49**, 2015–2020. 10.1107/S1600576716016514 PMC5139991 27980509

[bb43] Kalus, J., Neubauer, G. & Schmelzer, U. (1990). *Rev. Sci. Instrum.* **61**, 3384–3389.

[bb44] Khoury, C., Mensing, G. A. & Beebe, D. J. (2002). *Lab Chip*, **2**, 50–55. 10.1039/b109344d 15100862

[bb45] Kobayashi, I., Wada, Y., Uemura, K. & Nakajima, M. (2008). *Microfluid Nanofluid*, **5**, 677–687.

[bb46] Koester, S. & Pfohl, T. (2012). *Mod. Phys. Lett. B*, **26**, 1230018.

[bb47] Lang, P., Neiß, S. & Woias, P. (2011). *J. Micromech. Microeng.* **21**, 125024.

[bb48] Lee, J. N., Park, C. & Whitesides, G. M. (2003). *Anal. Chem.* **75**, 6544–6554. 10.1021/ac0346712 14640726

[bb49] Lee, J. S., Dylla-Spears, R., Teclemariam, N. P. & Muller, S. J. (2007). *Appl. Phys. Lett.* **90**, 074103.

[bb50] Lee, S.-J. J. & Sundararajan, N. (2010). *Microfabrication for Microfluidics.* Norwood: Artech House.

[bb51] Liberatore, M. W., Nettesheim, F., Wagner, N. J. & Porcar, L. (2006). *Phys. Rev. E*, **73**, 020504. 10.1103/PhysRevE.73.020504 16605317

[bb109] Lide, D. R. (2003). *CRC Handbook of Chemistry and Physics*, 84th ed. Boca Raton: CRC Press.

[bb84] LightFab (2018). Three-Dimensional Microfluidics, http://www.lightfab.de.

[bb53] Lin, Y.-S., Yang, C.-H., Wang, C.-Y., Chang, F.-R., Huang, K.-S. & Hsieh, W.-C. (2012). *Sensors*, **12**, 1455–1467. 10.3390/s120201455 PMC3304121 22438719

[bb54] Lindner, P. & Oberthür, R. (1984). *Rev. Phys. Appl.* (*Paris*), **19**, 759–763.

[bb55] Lopez, C. G., Watanabe, T., Martel, A., Porcar, L. & Cabral, J. T. (2015). *Sci. Rep.* **5**, 7727. 10.1038/srep07727 PMC4289890 25578326

[bb56] Lutz-Bueno, V., Kohlbrecher, J. & Fischer, P. (2015). *J. Non-Newt. Fluid Mech.* **215**, 8–18.

[bb57] Lutz-Bueno, V., Zhao, J., Mezzenga, R., Pfohl, T., Fischer, P. & Liebi, M. (2016). *Lab Chip*, **16**, 4028–4035. 10.1039/c6lc00690f 27713983

[bb58] Marmiroli, B. & Amenitsch, H. (2012). *Eur. Biophys. J.* **41**, 851–861. 10.1007/s00249-012-0843-3 22854870

[bb59] Martin, H. P., Brooks, N. J., Seddon, J. M., Luckham, P. F., Terrill, N. J., Kowalski, A. J. & Cabral, J. T. (2016). *Soft Matter*, **12**, 1750–1758. 10.1039/c5sm02689j 26739043

[bb60] McDonald, J. C. & Whitesides, G. M. (2002). *Acc. Chem. Res.* **35**, 491–499. 10.1021/ar010110q 12118988

[bb61] Meineke, G., Hermans, M., Klos, J., Lenenbach, A. & Noll, R. (2016). *Lab Chip*, **16**, 820–828. 10.1039/c5lc01478f 26862603

[bb62] Merlin, A., Angly, J., Daubersies, L., Madeira, C., Schöder, S., Leng, J. & Salmon, J.-B. (2011). *Eur. Phys. J. E*, **34**, 58. 10.1140/epje/i2011-11058-y 21674320

[bb63] Metwally, K., Robert, L., Queste, S., Gauthier-Manuel, B. & Khan-Malek, C. (2012). *Microsyst Technol.* **18**, 199–207.

[bb64] Metze, M., Barbe, S., Reiche, A., Kesting, A. & Schweins, R. (2017). *J. Neutron Res.* **19**, 177–185.

[bb65] Mildner, D. F. R. (2014). *J. Appl. Cryst.* **47**, 1247–1251.

[bb66] Møller, M., Nielsen, S. S., Ramachandran, S., Li, Y., Li, Y., Tria, G., Streicher, W., Petoukhov, M. V., Cerione, R. A., Gillilan, R. E. & Vestergaard, B. (2013). *PLoS One*, **8**, e74783. 10.1371/journal.pone.0074783 PMC3787022 24098668

[bb67] NIST (2017). *Neutron Activation and Scattering Calculator.* https://www.ncnr.nist.gov/resources/activation. NIST, Maryland, USA.

[bb110] NIST (2018). *Neutron Scattering Lengths and Cross Sections.* https://www.ncnr.nist.gov/resources/n-lengths. NIST, Maryland, USA.

[bb208] Norman, A. I., Zhang, W., Beers, K. L. & Amis, E. J. (2006). *J. Colloid Interface Sci.* **299**, 580–588. 10.1016/j.jcis.2006.02.025 16530780

[bb111] Osman, A., El-Sarraf, M., Abdel-Monem, A. & Abdo, A. E.-S. (2015). *Ann. Nucl. Energy*, **78**, 146–151.

[bb200] Papautsky, I., Brazzle, J., Swerdlow, H. & Frazier, A. B. (1998). *J. Microelectromech. Syst.* **7**, 267–273.

[bb69] Penfold, J., Staples, E., Tucker, I., Carroll, P., Clayton, I., Cowan, J., Lawton, G., Amin, S., Ferrante, A. & Ruddock, N. (2006). *J. Phys. Chem. B*, **110**, 1073–1082. 10.1021/jp051122m 16471644

[bb70] Pham, N., Radajewski, D., Round, A., Brennich, M., Pernot, P., Biscans, B., Bonneté, F. & Teychené, S. (2017). *Anal. Chem.* **89**, 2282–2287. 10.1021/acs.analchem.6b03492 28192906

[bb71] Pollack, L. & Doniach, S. (2009). *Methods Enzymol.* **469**, 253–268. 10.1016/S0076-6879(09)69012-1 20946793

[bb72] Poulos, A. S., Nania, M., Lapham, P., Miller, R. M., Smith, A. J., Tantawy, H., Caragay, J., Gummel, J., Ces, O., Robles, E. S. J. & Cabral, J. T. (2016). *Langmuir*, **32**, 5852–5861. 10.1021/acs.langmuir.6b01240 27196820

[bb112] Prince, E., Wilson, A. J. C., Hahn, T. & Shmueli, U. (1999). *International Tables for Crystallography*, Vol. C, *Mathematical, Physical and Chemical Tables*, ch. 2.6. Dordrecht: Kluwer.

[bb73] Qazi, S. J. S., Rennie, A. R., Tucker, I., Penfold, J. & Grillo, I. (2011). *J. Phys. Chem. B*, **115**, 3271–3280. 10.1021/jp108805m 21395302

[bb74] Rodriguez, M. A., Yang, P., Kotula, P. & Dimos, D. (2000). *Adv. X-ray Anal.* **43**, 332–337.

[bb75] Rodríguez-Ruiz, I., Radajewski, D., Charton, S., Phamvan, N., Brennich, M., Pernot, P., Bonneté, F. & Teychené, S. (2017). *Sensors*, **17**, 1266. 10.3390/s17061266 PMC5492703 28574461

[bb113] Root, J. H., Buyers, W. J., Page, J. H., Schaefer, D. W. & Brinker, C. (1989). *MRS Proc.* **166**, 379.

[bb76] Schindler, K. & Roosen, A. (2009). *J. Eur. Ceram. Soc.* **29**, 899–904.

[bb77] Schwemmer, F., Blanchet, C. E., Spilotros, A., Kosse, D., Zehnle, S., Mertens, H. D., Graewert, M. A., Rössle, M., Paust, N., Svergun, D. I., von Stetten, F., Zengerle, R. & Mark, D. (2016). *Lab Chip*, **16**, 1161–1170. 10.1039/c5lc01580d 26931639

[bb114] Sears, V. F. (1992). *Neutron News*, **3**(3), 26–37.

[bb78] Sharma, J., King, S. M., Bohlin, L. & Clarke, N. (2010). *Nucl. Instrum. Methods Phys. Res. A*, **620**, 437–444.

[bb79] Shastry, M., Luck, S. D. & Roder, H. (1998). *Biophys. J.* **74**, 2714–2721. 10.1016/S0006-3495(98)77977-9 PMC1299611 9591695

[bb80] Shibayama, M., Nagao, M., Okabe, S. & Karino, T. (2005). *J. Phys. Soc. Jpn*, **74**, 2728–2736.

[bb81] Shin, E., Choi, S.-H., Seong, B.-S., Lee, H.-C. & Lee, K. (2010). *Appl. Phys. A*, **99**, 621–625.

[bb82] Silva, B. F. (2017). *Phys. Chem. Chem. Phys.* **19**, 23690–23703. 10.1039/c7cp02736b 28828415

[bb83] Skou, M., Skou, S., Jensen, T. G., Vestergaard, B. & Gillilan, R. E. (2014). *J. Appl. Cryst.* **47**, 1355–1366. 10.1107/S1600576714012618 PMC4119951 25242913

[bb85] Sollier, E., Murray, C., Maoddi, P. & Di Carlo, D. (2011). *Lab Chip*, **11**, 3752–3765. 10.1039/c1lc20514e 21979377

[bb86] Squires, T. M. & Quake, S. R. (2005). *Rev. Mod. Phys.* **77**, 977–1026.

[bb87] Stehle, R., Goerigk, G., Wallacher, D., Ballauff, M. & Seiffert, S. (2013). *Lab Chip*, **13**, 1529–1537. 10.1039/c3lc41291a 23429654

[bb88] Takeda, M., Kusano, T., Matsunaga, T., Endo, H., Shibayama, M. & Shikata, T. (2011). *Langmuir*, **27**, 1731–1738. 10.1021/la104647u 21244071

[bb89] Tepe, T., Schulz, M., Zhao, J., Tirrell, M., Bates, F., Mortensen, K. & Almdal, K. (1995). *Macromolecules*, **28**, 3008–3011.

[bb90] Toft, K. N., Vestergaard, B., Nielsen, S. S., Snakenborg, D., Jeppesen, M. G., Jacobsen, J. K., Arleth, L. & Kutter, J. P. (2008). *Anal. Chem.* **80**, 3648–3654. 10.1021/ac800011y 18422341

[bb91] Tong, J., Nakajima, M., Nabetani, H., Kikuchi, Y. & Maruta, Y. (2001). *J. Colloid Interface Sci.* **237**, 239–248. 10.1006/jcis.2001.7461 11334539

[bb92] Trebbin, M., Steinhauser, D., Perlich, J., Buffet, A., Roth, S. V., Zimmermann, W., Thiele, J. & Förster, S. (2013). *Proc. Natl Acad. Sci. USA*, **110**, 6706–6711. 10.1073/pnas.1219340110 PMC3637725 23569240

[bb93] Tsao, C.-W., Chen, T.-Y., Woon, W. Y. & Lo, C.-J. (2012). *Microsyst. Technol.* **18**, 713–722.

[bb94] Vasudevan, M., Buse, E., Lu, D., Krishna, H., Kalyanaraman, R., Shen, A. Q., Khomami, B. & Sureshkumar, R. (2010). *Nat. Mater.* **9**, 436–441. 10.1038/nmat2724 20305641

[bb95] Vitale, A., Hennessy, M. G., Matar, O. K. & Cabral, J. T. (2015). *Adv. Mater.* **27**, 6118–6124. 10.1002/adma.201502607 26333100

[bb96] Weston, J. S., Seeman, D. P., Blair, D. L., Salipante, P. F., Hudson, S. D. & Weigandt, K. M. (2018). *Rheol. Acta*, **57**, 241–250.

[bb97] Whitesides, G. M. (2006). *Nature*, **442**, 368–373. 10.1038/nature05058 16871203

[bb98] With, S., Trebbin, M., Bartz, C. B., Neuber, C., Dulle, M., Yu, S., Roth, S. V., Schmidt, H.-W. & Förster, S. (2014). *Langmuir*, **30**, 12494–12502. 10.1021/la502971m 25216394

[bb99] Yearley, E. J., Sasa, L. A., Welch, C. F., Taylor, M. A., Kupcho, K. M., Gilbertson, R. D. & Hjelm, R. P. (2010). *Rev. Sci. Instrum.* **81**, 045109. 10.1063/1.3374121 20441370

[bb100] Yoo, M., Ogle, J., Borie, B., Lee, E. & Hendricks, R. (1982). *Acta Metall.* **30**, 1733–1742.

[bb101] Zhang, Y., Bottausci, F., Rao, M. P., Parker, E., Mezic, I. & Macdonald, N. (2008). *Biomed. Microdevices*, **10**, 509–517. 10.1007/s10544-007-9159-y 18214682

[bb102] Zheng, B., Roach, L. S. & Ismagilov, R. F. (2003). *J. Am. Chem. Soc.* **125**, 11170–11171. 10.1021/ja037166v 16220918

[bb103] Zhou, X., Li, J., Wu, C. & Zheng, B. (2008). *Macromol. Rapid Commun.* **29**, 1363–1367.

