# Supplementary material for: Microfluidic devices for small-angle neutron scattering
Source: J Appl Crystallogr. 2018 Jun 1;51(Pt 3):570–83. doi: 10.1107/S1600576718007264 (PMC5988002; doi:10.1107/S1600576718007264)
Supplement: Supplementary file 1 [file j-51-00570-sup1.pdf]

JOURNAL OF
APPLIED
CRYSTALLOGRAPHY

Volume 51 (2018)

Supporting information for article:

Microfluidic devices for small-angle neutron scattering

Carlos G. Lopez, Takaichi Watanabe, Marco Adamo, Anne Martel, Lionel Porcar and João T. Cabral

Journal of Applied Crystallography

ISSN 0021-8898

Supporting information: Microfluidic devices for Small Angle Neutron Scattering

Carlos G. Lopez,[a,†] Takaichi Watanabe,[a,‡] Marco Adamo,[a] Anne Martel,[b] Lionel Porcar[b] and Joao T. Cabral [a]*

[a]Department of Chemical Engineering, Imperial College London, South Kensington Campus, London SW7 2AZ, UK, [b]Institute Laue-Langevin, 71 avenue des Martyrs, 38042 Grenoble, France, [†]Present address: Institute of Physical Chemistry, RWTH Aachen University, Landoltweg 2, 52056 Aachen, Germany, and [‡]Present address: Department of Applied Chemistry, Okayama University, 3-1-1 Tsushima-naka, Kita-ku, Okayama, Japan. Correspondence e-mail: j.cabral@imperial.ac.uk

© 0000 International Union of Crystallography
Printed in Singapore – all rights reserved

1. Data reduction and calibration

Usual SANS experiments of liquids employ a measurement cuvette, often a quartz cell. The neutron beam impinges through the windows of the cuvette and illuminates a certain sample area, defined by the diaphragm, and thus a volume (by multiplication by the internal cuvette thickness). This arrangement is analogous to those shown in Figs 2b and d of the main paper, where the beam footprint is comparatively smaller than the microchannel width. The scattering vector is defined by:

$$q = \frac{4\pi}{\lambda} sin\left(\frac{\theta}{2}\right)$$

where λ is the neutron wavelength (typically 1-20 Å) and the scattering angle θ is defined in Figure S1; in the small angle limit, $q \approx 2\pi\theta/\lambda$. The scattering signal of a sample without a container, ignoring contributions from the empty beam and background noise, is simply the number of counts per unit time at a detector pixel with solid angle $\Delta\Omega$, given by

$$N_S(q) = \Phi(\lambda) A K t_S T_S \frac{d\Sigma_S}{d\Omega}(q)\Delta\Omega \tag{1}$$

where Φ is the neutron flux, A is the beam's cross sectional area, K is a calibration prefactor, t_s is the sample thickness, T_s is the sample transmission and $\frac{d\Sigma}{d\Omega}$ is the macroscopic differential scattering cross section. $\frac{d\Sigma}{d\Omega}(q)$ contains information about the sample's structure which the scattering experiment seeks to determine. Taking into account the contribution of a cell of uniform cross-section, as illustrated in Fig. 2b and d, the scattering from the sample reads

$$\frac{d\Sigma_S}{d\Omega} = \frac{1}{KA}\left(\frac{N_{S+W}}{t_s T_{S+W}} - \frac{N_W}{t_S T_W}\right) \tag{2}$$

calibrated in 'absolute' units of [cm^{-1}]. The subscript S refers to the sample, the subscript $S+W$ refers to the sample within a container (i.e. cell windows), and W the windows. Refinements of Eq 2 have been introduced (Brûlet *et al.*, 2007) to more rigorously account for the contribution of the sample container and geometry at large angles. In addition, neutron transmissions T are measured from the ratio of the neutron flux in the forward direction of the empty beam and that of the beam with the sample or cell. The scattered intensity recorded at the detector is thus broadly proportional to the irradiated volume (beam area × thickness) and inversely proportional to the transmission.

1.1. Data reduction in microfluidic SANS

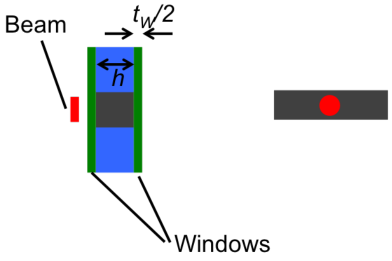

Figure S1
Standard cell configuration.

I. Conventional window configuration. As illustrated in Fig. 2 of the main text, a number of microdevice geometries can be employed in microfluidic SANS in terms of (i) the neutron beam illumination of the microchannels, (ii) effective illuminated sample volume, (iii) and number and type of laminated materials in the device. These have significant consequences to the correct reduction and calibration (to standard units of [cm^{-1}]) of SANS data for quantitative analysis. We first consider the 'standard' case, depicted in Fig. 2b and d, where the beam footprint is smaller than the lateral channel dimensions and traverses, therefore, two windows of the same material (e.g. glass or polymer), in addition to the liquid sample of interest. The conventional data reduction procedures apply to extract the scattering signal from the sample:

$$\frac{d\Sigma_S}{d\Omega} \equiv I_S[cm^{-1}] = \frac{1}{KA}\left(\frac{N_{S+W}}{hT_{S+W}} - \frac{N_W}{hT_W}\right) \tag{3}$$

where K is the calibration factor, measured by the direct beam flux, $T \equiv \Phi_i/\Phi_{empty}$ is transmission (the ratio of neutron flux Φ_i of specimen i with respect to the direct beam), and N the scattering per unit time and h the channel height. The indices

S, W and $S+W$ correspond to 'sample', 'window' and 'sample+window'. We use 'window' to refer to both windows. N_W and N_{S+W} have been corrected for the empty beam and background noise. The transmission of the empty microdevice is thus $T_{empty} \equiv T_W = e^{-\mu_W t_W}$, where μ_W is the neutron attenuation coefficient and t_W is the thickness of the window material. The transmission of the device filled with sample is simply $T_{S+W} = T_S T_W = e^{-\mu_S h} e^{-\mu_W t_W}$, where μ_S is the attenuation coefficient of the fluid sample. Transmissions are thus multiplicative, meaning that the transmission of the (sample + cell) is the product of the individual sample and cell transmissions.

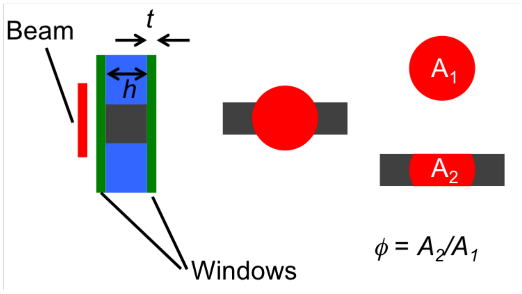

Figure S2
Overilluminated single channel configuration.

II. Overilluminated device of single component (e.g. glass). In the case that the beam overilluminates the channel(s), it is useful to define the area fraction that illuminates only the microdevice material ϕ, and the area that illuminates the microchannels $1-\phi$. The illuminated sample volume is thus $(1-\phi)h$. We consider first the case depicted in Fig 1(d), corresponding to a device made of a single material (e.g. glass or polymer). The experimentally measured transmission of the empty device is thus:

$$T_{Empty\ Device} = \phi e^{-\mu_W t_W} + (1-\phi) e^{-\mu_W (t-h)} \tag{4}$$

and the experimentally measured transmission of the sample-containing device reads

$$T_{S+Device} = \phi e^{-\mu_W t_W} + (1-\phi) e^{-\mu_W (t-h)} e^{-\mu_S (h)} \tag{5}$$

The total measured scattering signal has thus two (additive) contributions:

$$N_{S+Device} = \phi N_W + (1-\phi) N_{S+W} \Leftrightarrow N_{S+W} = \frac{N_{S+Device} - \phi N_W}{1-\phi} \tag{6}$$

and therefore, the scattering intensity from the sample can be obtained as

$$I_S = \frac{1}{KA} \left(\frac{N_{S+W}}{h T_{S+W}} - \frac{N_W}{h T_W} \right) \tag{7}$$

The challenge with eq. (7) is that N_{S+W}, T_{S+W} and T_W are not directly measured experimentally. Instead, the overall T and $I_{S+Device}$, and T and $I_{empty/device}$ are measured. These must therefore be estimated from the experimental data. However, many simplifications are usually possible to an accuracy of 1% or better, for instance when $t-h \simeq t$ (and thus $T_{Empty\ Device} \simeq T_W$ and $N_{Empty\ Device} \simeq N_W$) or when $N_{S+W} \gg N_W$, yielding

$$I_S = \frac{1}{KA} \left(\frac{\frac{N_{S+Device} - \phi N_{Empty\ Device}}{1-\phi}}{h T_{S+W}} - \frac{N_{Empty\ Device}}{h T_{Empty\ Device}} \right) \tag{8}$$

where

$$T_{S+W} = \frac{T_{measured} - \phi T_{Empty\ Device}}{1-\phi} \tag{9}$$

which can now all be measured directly.

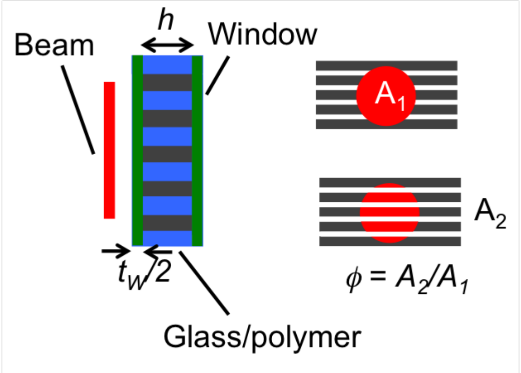

Figure S3
Overilluminated multichannel reduction.

III. Overilluminated window and matrix sandwich. The configuration shown in Fig 1(c), depicting an overilluminated channel (or channels) or a microdevice comprising two windows (e.g. glass) and a patterned channel matrix (e.g. NOA81) requires a similar treatment. In this case,

$$T_{Empty\ Device} = \phi e^{-\mu_W t_W} e^{-\mu_P t_P} + (1-\phi) e^{-\mu_W t_W} \tag{10}$$

where t_W and t_P are the thicknesses of the window and polymer layers, and μ_P the attenuation coefficient of the polymer. In the presence of the fluid sample:

$$T_{S+Device} = \phi e^{-\mu_W t_W} e^{-\mu_P t_P} + (1-\phi) e^{-\mu_W t_W} e^{-\mu_S h} \tag{11}$$

As above, the measured scattering intensity can be written as

$$N_{S+Device} = \phi N_{P+W} + (1-\phi) N_{S+W} \Leftrightarrow N_{S+W} = \frac{N_{S+Device} - \phi N_{P+W}}{1-\phi} \tag{12}$$

and therefore, the scattering intensity from the sample can be obtained as

$$I_S = \frac{1}{KA} \left(\frac{N_{S+W}}{h T_{S+W}} - \frac{N_W}{h T_W} \right) \tag{13}$$

where N_{S+W} is given by the equation above. For these types of devices, it is generally feasible (and preferable) to measure experimentally I_W, T_W (e.g. two glass plates) and I_{P+W}, T_{P+W} (e.g. an area of the device with no patterned microchannels). Otherwise, the procedure (and simplifications) discussed in (ii)

can also be employed. Examples of some of the above procedures have been applied to reduce microfluidic-SANS data in earlier studies, see references (Adamo *et al.*, 2017; Adamo *et al.*, 2018)

2. Estimations of background and transmission

As discussed in the main text, an optimal neutron cell should exhibit low brackground scattering and high transmission. We next outline some basic theory that allows us to estimate how different elements will perform for these two requirements. The differential scattering cross section for an ensemble of N nuclei with scattering lengths b is given by:

$$\frac{d\sigma}{d\Omega} = \sum_{i,j}^{N} b_i b_j \langle e^{i\mathbf{qR_{i,j}}}\rangle = \{b\}^2 \sum_{i,j}^{N}\langle e^{i\mathbf{qR_{i,j}}}\rangle + \sum_{i,j}^{N}\delta b_i^2 = \left[\frac{d\sigma}{d\Omega}\right]_{coh} + \left[\frac{d\sigma}{d\Omega}\right]_{inc} \quad (14)$$

where $\langle ...\rangle$ denotes an ensemble average over the nuclei positions, $\{\}$ an average over the different nuclei, $b_i = \{b\} + \delta b_i$, N is the total number of isotopes in the sample, $\mathbf{q}$ is the scattering vector and $\mathbf{R_{i,j}}$ is the vector joining the position of scattering species i and j. The subscripts '*coh*' and '*inc*' refer to coherent and incoherent scattering respectively. The former yields information about spatial correlations in a sample.

The macroscopic differential scattering cross section $\frac{d\Sigma}{d\Omega}$ (usually expressed in 'absolute' units of cm^{-1}), is referred to as the scattering intensity (I), and can be obtained my multiplying eq. 14 above by the number density of scatterers N/V. Incoherent scattering is isotropic and therefore evenly distributed over the entire solid angle

$$I_{inc} \equiv \frac{d\Sigma_{inc}}{d\Omega} = \frac{N}{V}\frac{1}{4\pi}\sigma_{inc} \quad (15)$$

and Σ_{inc} and σ_{inc} are the total macroscopic and microscopic scattering cross-sections respectively. The high q scattering signal generally contains a coherent and incoherent contribution. Some materials, for example D_2O, scatter coherently in the high q region that is nearly q independent, since density fluctuations occur at much smaller lengthscales than the distances probed even at large scattering angles. Thus, the D_2O signal in the $\simeq$ 0.01 - 0.5 $Å^{-1}$ range is $I \simeq 0.05$ cm^{-1} while $I_{inc} \simeq 0.015$ cm^{-1} (Hammouda, 2008): the coherent part of scattering dominates even in the high q region where I becomes q-independent. This coherent contribution can be important for a number of microfabrication materials, notably glasses (although its computation from pair-correlations functions is complex, in particular for amorphous materials, including inorganic glasses and polymers). I_{inc} therefore acts as a lower bound estimate of a material's quality in the high q region ($\simeq$ 0.1 - 0.5 $Å^{-1}$). Although the I_{coh}(q) profile will depend on the atomic and molecular arrangement at this scale, a number of assumptions can be made to estimate the quality of a window-material for a SANS experiment.

We estimate the 'background scattering' $I_{background}$, assuming all coherent scattering is purely isotropic and therefore:

$$I_{background} = \frac{N}{V}\frac{1}{4\pi}\sigma_{coh}(1 - f_{Cr}) + I_{inc} \quad (16)$$

where f_{Cr} is the crystalline fraction of the material. The inclusion of the term in brackets assumes the crystalline fraction of the material does not scatter coherently in the SANS region. The first term on the RHS of eq. 16 represents an upper limit to the real coherent contribution at high q values as the scattering signal is never fully isotropic: it generally increases as q decreases for low q values due to large scale inhomogeneities. Additionally, amorphous materials display broad correlation peaks at very high q (outside the window of interest, e.g. quartz at 1.55 $Å^{-1}$ (Root *et al.*, 1989)) due to short range order. I_{inc} and $I_{background}$ act thus as lower and upper bounds to the experimental background intensity respectively.

Another key indicator for material suitability for SANS experiment is the neutron transmission T, i.e. the ratio of incident to transmitted neutron fluxes in the forward direction.

$$T = T^{abs}T^{scatt} = e^{-(\mu_{abs}(\lambda)+\mu_{scatt})t} \quad (17)$$

where T^{abs} takes into account absorbed neutrons and T^{scatt} scattered neutrons. The absorption coefficient is $\mu_{abs}(\lambda) = \sum_i \rho_i \frac{\lambda}{1.8}\sigma_{abs}(1.8Å)$, where ρ_i is the number density of the ith component of the material, λ is the neutron wavelength and $\sigma_{abs,i}(1.8Å)$ is the absorption cross section of the ith component for $\lambda = 1.8$ Å. The scattering coefficient is $\mu_{scatt} = \sum_i \rho_i(\sigma_{inc,i} + \sigma_{coh,i})$, where $\sigma_{inc,i} + \sigma_{coh,i}$ are the coherent and incoherent microscopic cross sections for the composite material. Materials with T $\simeq$ 1 effectively do not absorb or scatter neutrons, and are therefore excellent window materials, while materials with low T either absorb or scatter strongly, hence decreasing the effective flux or increasing the background signal, and are thus undesirable.

Values for σ_{coh} and σ_{inc} for specific isotopes or elements are tabulated (NIS, 2018; Sears, 1992) and, the coherent and incoherent cross sections for mixtures of isotopes can be calculated according simple formulae:

$$\sigma_{coh}(A_aB_bC_c...) = \sqrt{4\pi}(f_A\sqrt{\sigma_{coh,A}}+f_B\sqrt{\sigma_{coh,B}}+f_C\sqrt{\sigma_{coh,C}}+...)^2$$

$$\sigma_{inc}(A_aB_bC_c.) = f_A\sigma_{inc}(A) + f_B\sigma_{inc}(B) + f_C\sigma_{inc}(C) + ...$$

where f_i is the number fraction of the ith element.

2.1. Overview of neutron-elemental interactions and candidates for microfabrication materials

Figure 1 plots the relevant neutron quantities for significant elements relevant for microdevices. We consider the 50 most abundant elements, excluding rare earth, noble gases and highly reactive elements, but including major industrial elements and common precious metals (Ag and Au). We take $\rho = 1$ g/cm^3 for gaseous elements at room temperature, as approximately valid for polymeric devices. The quantities plotted represent the average isotopic abundance for all elements except hydrogen, which is split into ^1H and ^2H. As seen in Figure 1a, most low atomic

number (Z $\lesssim$ 40) elements yield minimal neutron absorption, except for lithium, boron and cobalt. In addition hydrogen scatters strongly due to its large incoherent cross section, and thus its content must be minimised. Figure 1b shows the transmissions for 1 mm (black) and 0.1 mm (red lines) slabs of the respective elements. As a reference, a common Hellma QS-series (2×1 mm windows of Suprasil quartz) exhibits T$\simeq$ 96 % (at λ=6Å).

The incoherent scattering intensity I_{inc} and $I_{background}$ (with f_{Cr} = 0) are plotted in Fig 4(c), with hydrogen standing out for it large I_{inc}. The composition of various glasses are compiled in Table S1. With the exception of boron containing materials, most common glasses yield high neutron transmissions and low background scattering. Metals yield reasonably low I_{inc}, with the stronger incoherent scatterers such as cobalt or nickel still showing an I_{inc} lower than the scattering of D_2O ($\simeq$ 0.05 cm^{-1}, a relatively weak scatterer) in the 0.01 - 0.5 $Å^{-1}$ q window.

Most non-hydrogenous materials should thus yield reasonably high quality neutron cells (T $\gtrsim$ 96%) in the high q region (except the strong absorbers discussed above). Further exceptions include crystalline materials with characteristic repeat distances $d \geq$ 12-15 Å, which display a strong structural peak around $q^* = 2\pi/d$ where d is the characteristic spacing between scattering centres.

The considerations presented above allow us to: 1) discard certain materials based on their strong neutron absorption, 2) estimate their quality as high q window materials and 3) estimate their generally suitability based on their transmission. Generally, (single) crystal materials should be favoured (provided the crystalline peaks lie outside of the SANS region, which usually holds (Prince *et al.*, 1999)) as the coherent contribution to scattering is confined to a series of intense, sharp peaks. Polycrystalline materials will scatter strongly in the low q region due to grain boundary interfaces and are generally isotropic. Hydrogen, boron or cadmium containing materials should be avoided due to strong incoherent (hydrogen) and strong absorption (boron and cadmium along with some heavy elements). The effect on temperature and neutron wavelength as well as inelastic effects (Glinka, 2011) may become important at high q, depending on measurement conditions.

3. Composition of inorganic glasses

Table 1 compiles the composition and scattering properties of several commercially available glasses. With the exception of borosilicate and aluminosilicate glass, which show significantly higher absorption due to the boron content, all other types of glass yield similar neutron properties. Any of the glasses examined (even borosilicate, as it is commercially available at low cost in thin sheets(Lopez *et al.*, 2015)) are good window materials for SANS.

Component (wt%)	Borosilicate glass	Soda lime glass	Aluminosilicate glass*	*Fused silica*	Lead glass**
SiO_2	81	73	55	100	73(100-x)
Na_2O	4	14	1		14(100-x)
Al_2O_3	2		10.4		0.1(100-x)
B_2O_3	13		7		
MgO		4			4(100-x)
CaO		9	21		9(100-x)
Fe_2O_3		0.1			0.1(100-x)
PbO_2					x
T^a/mm	0.28	1.00	0.45	1.00	1.00
T^{sc}/mm	0.97	0.97	0.98	0.98	0.97
S_{inc} (cm^{-1})	0.00086	0.00091	0.00051	0.00001	0.00087
S_{bckg} (cm^{-1})	0.018	0.018	0.018	0.015	0.019
S_{back}^{exp} (cm^{-1})	0.006	0.0035		0.0035	

Table 1

Composition, calculated transmissions per mm, calculated scattering intensities, and measured background intensities for different types of glass. *composition taken from Aldrich catalogue, the list of components given adds up to 90% and they have been rescaled by a factor of $1\frac{1}{9}$. ** x represents the weight fraction of lead oxide (Osman *et al.*, 2015), which typically varies between 2-77% depending on application (James & Doremus, 2008). Calculations for x = 0.25.

4. Transmission of microfabrication materials

	T/mm	Device thickness (mm)	T_d	t_{96} (mm)
Aluminium	0.99	5.00	0.97	7.16
Copper*	0.91	0.4[a]	0.96	0.41
Steel*	0.62	0.4[a]	0.83	0.09
Nickel*	0.81	0.02	1.00	0.19
PDMS	0.60	1.00	0.60	0.08
Polyethylene[a]	0.45	0.40	0.73	0.05
Polystyrene	0.62	0.40	0.82	0.08
PMMA*	0.61	0.40	0.82	0.08
Polycarbonate*	0.70	0.40	0.87	0.11
Thiolene	0.57	0.40	0.80	0.07
Kapton	0.66	0.10	0.96	0.10
d-PS*	0.93	0.40	0.97	0.56
d-PMMA*	0.93	0.40	0.97	0.56
Borosilicate glass	0.41	0.28	0.78	0.05
Soda lime glass	0.98	2	0.94	1.74
Quartz	0.98	0.88	0.98	1.80
Fused silica	0.98	2.30	0.94	1.62
Silicon	0.99	2.00	0.98	5.21
Hellma Cell	0.98	2.00	0.96	2.00

Table 2

Transmission parameters for various microfabrication materials. Materials marked with $*$ are calculated values. T/mm is the transmission for a slab of 1 mm thickness, T_d the transmission of a device (see methods section) and t_{96} the thickness of the material which gives 96% transmission. [a] estimated as the same thickness required for polymeric devices, this probably represents an upper bound.

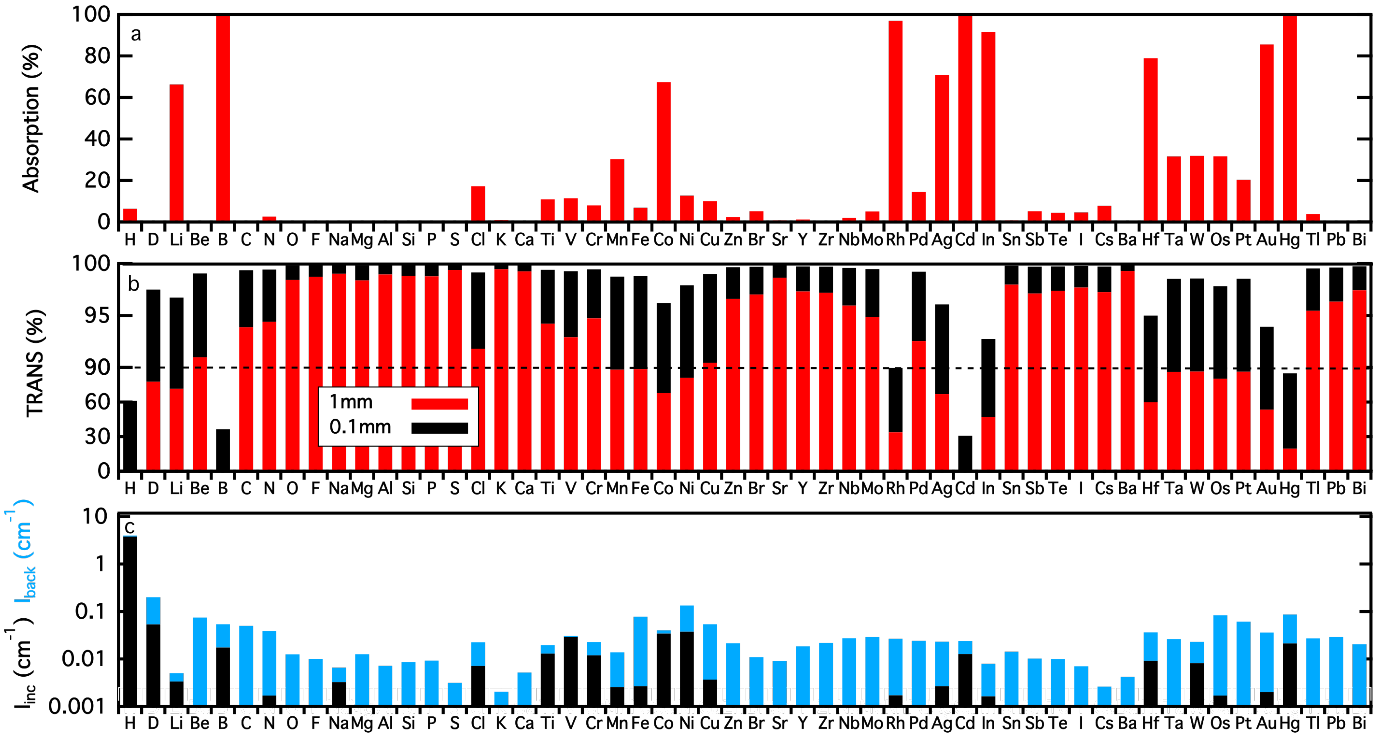

Figure S4

Key neutron scattering properties for the 50 most abundant elements, excluding rare earth and highly reactive elements, and including major industrial elements and precious elements Ag and Au, computed from tabulated scattering cross sections(NIS, 2018) and densities (Lide, 2003). Hydrogen is split into Protium (^1H) and deuterium (^2H or D) All values are for neutron wavelength $\lambda = 6$ Å. For elements which are gases at room temperature, such as hydrogen, we select ρ = 1 g/cm^3, discussed in the text. a) Percentage neutrons absorbed ($(1 - T_a)\times100$) when travelling through 1mm of a given element . b) Neutron transmission: the red bars indicate the transmission for d = 1 mm and the black bars indicate the transmission for d = 0.1 mm. Note the vertical axis changes scale at T = 90. c) red bars: I_{inc}, black bars I_{back}.

5. Pumps

Microflows may be passively generated via capillary forces (Dolník *et al.*, 2000) or actively, by the means of volume or pressure driven pumps. In the latter, the flowrate is set by a dispensed volume over time, which may be achieved by linear displacement pumps, in which a linear movement of the syringe is translated to a well-known infused volume, peristaltic pumps, in which a rotor is used to locally deform the tubing and thus push a defined volume of fluid, or by pressure driven pumps, which ensure a constant pressure in the microfluidic device, regardless of fluidic resistances in the system.

Peristaltic pumps (e.g. Braintree BS-900) are useful to create a closed fluid-loop circulating at relatively high flowrates (2.8 mL/h to 0.900 mL/min). A major drawback is that the flowrate is characterised by oscillations of the order of 3%, according to the geometry of the rotor. For these reasons, their use in microfluidics is marginal and limited to specific operations.

Displacement syringe pumps translate a linear translation into a volume driven flow. The accuracy of this system is related to the precision of the driving motor. Specifically, the motor moves in discrete (micro-)steps, which determine the minimal amount of fluid that can be dispensed. Generally, the achievable flowrates are in the order of 10s of pL/min to 200 mL/min, depending on system and syringe piston. For inexpensive systems (e.g. Braintree BS8000, Harvard PHD2000), the accuracy is around 1% of the flowrate and pulses become more visible at low flowrates. By accurately choosing the motor and the gear system it is possible to improve the accuracy to 0.25% (Harvard 33 DDS). The advantages of displacement pumps are that the setup of experiments is simple and relatively inexpensive, and the fluid volumes are well known. However, the response time of the systems (typically $\sim$ 1 s) depends strongly on the compliance of the components (syringe, tubing, microfluidic chip), the flow is somewhat pulsed (especially at low flowrates) and the pressure is not controlled, which can cause the pumps to stall.

Pressure driven pumps allow faster response times, of the order of 10s of ms (Elveflow OB1 MK3), high stability and pulseless flow in a pressure range of typically 0 to 10 bar (Elveflow-OB1 MK3, Dolomite microfluidics-Mitos P-Pump). The accuracy of this setup can reach values around 5×10^{-3} %, but the experimental setups are more complicated and the pumps are more expensive.

References

(2018). Neutron scattering lengths and cross sections. Https://www.ncnr.nist.gov/resources/n-lengths/.

Adamo, M., Poulos, A., Miller, R. M., Lopez, C. G., Martel, A., Porcar, L. & Cabral, J. T. (2017). *Lab on a Chip*, **17**, 1559–1569.

Adamo, M., Poulos, A. S., Lopez, C. G., Martel, A., Porcar, L. & Cabral, J. T. (2018). *Soft Matter*.

Brûlet, A., Lairez, D., Lapp, A. & Cotton, J.-P. (2007). *Journal of Applied Crystallography*, **40**(1), 165–177.

Dolník, V., Liu, S. & Jovanovich, S. (2000). *Electrophoresis*, **21**(1), 41–54.

Glinka, C. J. (2011). *Journal of Applied Crystallography*, **44**(3), 618–624.

Hammouda, B. (2008). *Natl. Institute Standards Technology Center for Neutron Research, Gaithersburg, MD*.

James, F. & Doremus, R. (2008). *Ceramic and glass materials: Structure, properties and processing*. London: Springer.

Lide, D. R. (2003). *CRC Handbook of Chemistry and Physics, 84th Edition*. CRC Press.

Lopez, C. G., Watanabe, T., Martel, A., Porcar, L. & Cabral, J. T. (2015). *Scientific Reports*, **5**, 7727.

Osman, A., El-Sarraf, M., Abdel-Monem, A. & Abdo, A. E.-S. (2015). *Annals of Nuclear Energy*, **78**, 146–151.

Prince, E., Wilson, A. J. C., Hahn, T. & Shmueli, U. (1999). *International tables for crystallography*. International Union of Crystallography.

Root, J. H., Buyers, W. J., Page, J. H., Schaefer, D. W. & Brinker, C. (1989). In *MRS Proceedings*, vol. 166, p. 379. Cambridge Univ Press.

Sears, V. F. (1992). *Neutron news*, **3**(3), 26–37.
